# The pale spear‐nosed bat: A neuromolecular and transgenic model for vocal learning

**DOI:** 10.1111/nyas.14884

**Published:** 2022-09-07

**Authors:** Sonja C. Vernes, Paolo Devanna, Stephen Gareth Hörpel, Ine Alvarez van Tussenbroek, Uwe Firzlaff, Peter Hagoort, Michael Hiller, Nienke Hoeksema, Graham M. Hughes, Ksenia Lavrichenko, Janine Mengede, Ariadna E. Morales, Maximilian Wiesmann

**Affiliations:** ^1^ School of Biology University of St Andrews St Andrews UK; ^2^ Neurogenetics of Vocal Communication Group Max Planck Institute for Psycholinguistics Nijmegen The Netherlands; ^3^ TUM School of Life Sciences Technical University of Munich Freising Germany; ^4^ Neurobiology of Language Department Max Planck Institute for Psycholinguistics Nijmegen The Netherlands; ^5^ LOEWE Centre for Translational Biodiversity Genomics, Faculty of Biosciences, Senckenberg Research Institute, Goethe‐University Frankfurt Germany; ^6^ School of Biology and Environmental Science University College Dublin Belfield Ireland; ^7^ Department of Medical Imaging Anatomy Radboud University Medical Center, Donders Institute for Brain, Cognition & Behavior, Center for Medical Neuroscience, Preclinical Imaging Center PRIME, Radboud Alzheimer Center Nijmegen The Netherlands

**Keywords:** bats, genome, language, MRI, Phyllostomus discolor, speech, tracing, vocal production learning

## Abstract

Vocal learning, the ability to produce modified vocalizations via learning from acoustic signals, is a key trait in the evolution of speech. While extensively studied in songbirds, mammalian models for vocal learning are rare. Bats present a promising study system given their gregarious natures, small size, and the ability of some species to be maintained in captive colonies. We utilize the pale spear‐nosed bat (*Phyllostomus discolor*) and report advances in establishing this species as a tractable model for understanding vocal learning. We have taken an interdisciplinary approach, aiming to provide an integrated understanding across genomics (Part I), neurobiology (Part II), and transgenics (Part III). In Part I, we generated new, high‐quality genome annotations of coding genes and noncoding microRNAs to facilitate functional and evolutionary studies. In Part II, we traced connections between auditory‐related brain regions and reported neuroimaging to explore the structure of the brain and gene expression patterns to highlight brain regions. In Part III, we created the first successful transgenic bats by manipulating the expression of *FoxP2*, a speech‐related gene. These interdisciplinary approaches are facilitating a mechanistic and evolutionary understanding of mammalian vocal learning and can also contribute to other areas of investigation that utilize *P. discolor* or bats as study species.

## Graphical abstract

Vocal learning is a key trait in the evolution of speech. Mammalian models for vocal learning are rare, and bats present a promising study system. We utilized the pale spear‐nosed bat to generate new, high‐quality genome annotations, examine structure and gene expression patterns in the brain, and created the first successful transgenic bats by manipulating expression of *FoxP2*, a speech‐related gene.

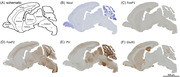

## INTRODUCTION

Vocal production learning (herein vocal learning) is the ability to produce modified or novel vocalizations, as a result of learning from the experience of the acoustic signals of others.^1^, ^2^ This ability is a key component of how humans learn the vast repertoire they use in speech and is employed by only a small number of other animals (selected birds, whales, dolphins, seals, bats, and elephants).^3^, ^4^ The spread of this trait across such evolutionarily diverse species suggests its evolution multiple times in the animal kingdom. There is good evidence that at least some of the mechanistic underpinnings of this trait are convergent across these vast evolutionary distances. For example, in humans, mutation of the *FOXP2* a gene causes a severe childhood disorder of speech,^5^ while in songbirds, FOXP2 manipulation interferes with vocal (song) learning.^6^, ^7^ This suggests that comparative approaches are likely to reveal fundamental mechanisms underlying the biology and evolution of vocal learning in animals, as well as shedding light on human speech and language.^8^

In recent years, bats have received increased attention as model systems for studying speech and language‐related traits such as vocal learning. We have previously outlined the benefits of employing these animals and the tools that would be needed to make them a powerful system for revealing biological and evolutionary mechanisms of vocal learning.^9^, ^10^ Multiple bat species are thought to be vocal learners, with evidence documented in subfamilies across Chiroptera.^11^ This pattern is consistent with the early evolution of vocal learning in bats,^11^ although much more evidence is needed to explore this hypothesis. Evidence for vocal learning in bats comes from a range of vocal behaviors, including modification of echolocation calls as well as social calls used for purposes, such as parent–offspring reunions, territorial defense, courtship, and group cohesion. In species from the Rhinolophidae and Hipposideridae families, there is evidence of learned modification of echolocation calls.^12^, ^13^ Promising work on bat vocal learning comes from multiple families. Juvenile Egyptian fruit bats (*Rousettus aegyptiacus*) were shown to modify call frequencies toward playbacks, and their call development is abnormal in isolated situations in which juveniles were housed with mothers who were rarely producing social calls.^14^, ^15^ Juvenile male sac‐winged bats (*Saccopteryx bilineata*) learn territorial and courtship songs from adult males in their environment.^16^ In addition, these juveniles show repetitive and variable vocal behaviors during learning phases that show striking parallels with human babbling during infant speech learning periods.^17^ Lastly, in species from the family Phyllostomidae, there is evidence for juvenile and adult modification of social calls. In the greater spear‐nosed bats (*Phyllostomus hastatus)*, adult calls that seem to denote group identity were modified following the experimental transfer of individuals between groups.^18^ In the pale spear‐nosed bat (*Phyllostomus discolor)*, juvenile isolation calls used by pups to interact with their mothers were modified to be more similar to computer playbacks in hand‐reared individuals.^19^ Furthermore, our own work has demonstrated that *P. discolor* bats that were deaf from an early age had a modified vocal repertoire suggesting some reliance on hearing conspecific vocalizations to produce the appropriate repertoire.^20^ We have also shown via operant conditioning paradigms that *P. discolor* bats were able to modify the properties of their social calls as adults.^21^, ^22^

Herein, we aim to outline the progress that has been made in our own work to establish the *P. discolor* bat as a tractable species for studying the neurogenetic mechanisms underlying learned vocal communication. *P. discolor* has several features that recommend it for in‐depth study of the neurogenetic underpinnings of vocal learning. They are small, predominantly frugivorous (with additional insectivore foraging), and thrive in captive breeding colonies.^23^ There is a rich history of neuroethological research in this species from which we can benefit and on which we can build—particularly since much of the previous research has explored the perception and production of vocalizations in the context of echolocation behavior.^24^, ^25^, ^26^, ^27^, ^28^, ^29^, ^30^, ^31^, ^32^ Furthermore, these are highly social animals with a complex vocal repertoire that they use for social interaction. The social calls of this species are dissimilar in frequency, duration, and structure to their echolocation calls, making them easily distinguishable.^33^, ^34^, ^35^

Our ultimate goal is to be able to understand the evolution and mechanistic underpinnings of learned vocal behavior, which necessitates an examination of the behavior as well as the underlying genetics and neurobiology. We have previously described our developments in behavioral aspects of vocal learning in *P. discolor*, including reporting their vocal repertoire in a social context,^33^ the effect of early deafening on repertoire,^20^ and controlled paradigms for testing vocal usage and vocal production learning in isolation.^21^, ^22^ Rather than revisiting these studies, we here focus on new advances made in the development of genomic, molecular, and neurobiological tools and approaches. With this, we have laid some foundations for understanding bat vocal learning from genes, to brains, to behavior. This neurogenetic model of mammalian vocal learning will allow us to make parallels with other mammals (including humans) and with birds to reveal biological and evolutionary mechanisms that underlie vocal learning, and ultimately factors that shaped the evolution of speech and language.

## RESULTS

### Part I: Genomics

Reference quality genomes are important for studying genotype–phenotype relationships, the molecular mechanisms underlying phenotypes, the evolution of traits, and for conservation efforts.^36^ Genomes are generally considered reference quality when almost all sequences can be mapped onto known chromosomes when those chromosomes are highly contiguous (i.e., few gaps are present), and when few sequence errors are present.^37^ ^,^ b For reference quality genomes to be useful, they must also be carefully annotated to define gene coding regions and noncoding elements. Only with good quality gene annotations can the (often) billions of nucleotides in the genome make sense during functional, molecular, or evolutionary studies. Determining the quality of annotations is challenging, but one way this can be done is to assess the percentage completeness for a set of highly conserved genes (e.g., BUSCO score).^38^ Reference quality genomes are only recently becoming the norm thanks to large‐scale genome projects, such as the Earth Biogenome Project,^39^ the Vertebrate Genome Project,^37^ and the Bat1K Genome Project—which aims to sequence all living bat species.^36^ Reference quality annotated bat genomes generated as part of the Bat1K project are now facilitating in‐depth investigations into research avenues as diverse as host–virus interactions, cancer, healthy aging, and echolocation.^40^

The genome of the *P. discolor* bat was originally published in 2020 by the Bat1K consortium as part of our release of the first reference quality bat genomes.^40^ This genome (chromosome 2n = 32) is 2.095 Gb in size, has an average QV of 42.9, and is assembled into 41 scaffolds (scaffold N50 = 171.08 Mb). The original assembly that was released with this genome identified 20,953 genes and found 96.8% BUSCO gene completeness (0.3% fragmented, 2.9% missing). At that time, these annotated genomes represented some of the best for mammals other than humans and mice.^40^ We subsequently found via manual inspection that several gene models were incomplete or missing, demonstrating that these annotations could still be improved. Given the importance of good quality annotations for accurate interpretation of findings from large‐scale evolutionary and omics approaches, we sought to improve the annotations of both coding and noncoding regions of the *P. discolor* genome.

#### Gene annotations

**FIGURE 1:**
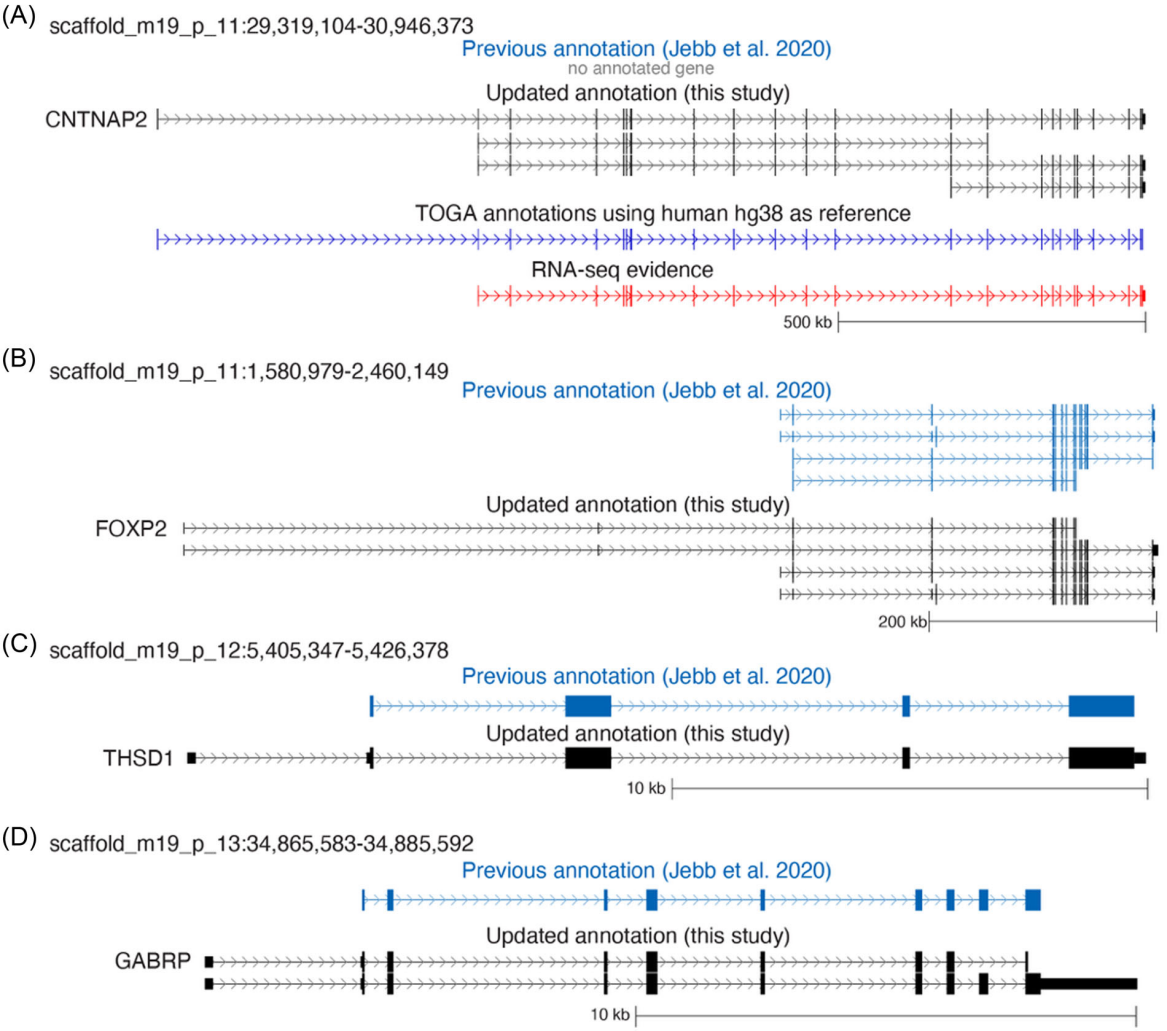
Improved gene annotations of the *P. discolor* genome. UCSC genome browser screenshots show examples of loci with various improvements, including annotation of (A) a gene previously missing from the annotations; *CNTNAP2*, (B) new exons; *FOXP2*, (C) improved UTRs; *THSD1*, and (D) alternative isoforms; *GABRP*. In each panel, the top track (light blue) indicates the previous annotation as reported in Jebb *et al.* 2020, and the second track (in black) reports the updated annotation from the current study. Additional tracks in blue and red depict experimental evidence to support the current annotation. Horizontal lines indicate the predicted or observed genetic locus. Vertical lines or thick rectangles indicate the exons identified via predictions or functional data. Thinner rectangles indicate untranslated regions (UTRs) that extend out from the first exon (5’UTR) or the last exon (3’UTR). Arrows indicate a noncoding sequence (introns) between coding regions (exons) and the direction of coding in the genome. Scale bars are indicated below each gene in kilobases (kb).

To improve the *P. discolor* annotations, we produced additional functional (transcriptomic) data from a range of tissues (Table S1), applied a refined version of the TOGA annotation pipeline (Kirilenko *et al.*, In review; https://github.com/hillerlab/TOGA Version 1.0), and reprocessed ISOseq data^40^ with a strategy that allowed us to prioritize transcripts with known splice‐sites and filter lower‐quality transcripts (see Supplementary Material for Methods). Together, these steps improved upon previous annotation strategies and addressed potential issues that prevented us from annotating some genes. Using the latest BUSCO odb10 mammalia dataset to assess annotation completeness,^38^ we produced a marked improvement in completely detected BUSCO genes—from 96.8% to 99% (Table 1), indicating that most previously missed conserved genes are now annotated (see Figure 1A for an example of annotation of a gene that was previously missing from the annotations). Furthermore, the total number of genes annotated increased from *N* = 21,516 to *N* = 25,058 (Table 1). This also produced an increase in the untranslated regions (UTRs) that were annotated, providing more complete gene models for many loci (Figure 1B–D). The new *P. discolor* genome annotation (File S1) represents one of the most comprehensive annotations of any bat genome to date and will facilitate large‐scale omics approaches in this species to understand the genetic mechanisms underlying complex behavioral traits, including but not limited to vocal learning. In addition, the quality of the annotations also gives confidence when employing evolutionary genomics approaches to answer a range of questions across other fields.

**TABLE 1:** New *P. discolor* genome annotation metrics

	Previous annotation	New annotation
BUSCO—complete	96.80%	99.00%
BUSCO—fragmented	0.30%	0.20%
BUSCO—missing	2.90%	0.80%
Number of genes	21,516	25,058
Number of transcripts	62,971	72,323
Transcripts with 5’ UTR	31,311	53,021
Transcripts with 3’ UTR	31,305	53,831
Average number of exons	11	10
Average length of CDS	45,525	40,484

BUSCO values based on dataset mammalia_odb10.

##### Noncoding annotations: miRNAs and 3’UTRs

Protein‐coding regions often represent less than 2% of the sequence of mammalian genomes.^41^ Noncoding regions have important functions in regulating gene and protein expression levels^42^, ^43^ making it crucial to annotate noncoding regions to understand complex traits and their evolution. However, annotating noncoding regions is particularly challenging given their variability in sequence, differing functions across tissues, and the relative lack of functional data compared to protein‐coding regions.^44^, ^45^ We previously annotated noncoding RNAs in the *P. discolor* genome and five other bat species, reporting similar representations of noncoding RNA classes as found in other mammals.^40^ Because of the important role that microRNAs play in regulating protein expression,^43^ we have now focused on refining the annotation of miRNAs in *P. discolor* and the noncoding 3’UTR regions they target. To improve upon the miRNA annotations, we devised an annotation approach that builds on that of miRanalyzer.^46^ Our approach relied on sequence homology for the annotation of miRNAs across species (Table S2) and incorporated newly generated small RNA sequencing data from multiple bat tissues (testes, liver, cortex, and striatum) across five individuals, plus kidney data from one individual. To improve 3’UTR annotation, we performed MACE sequencing using the same testes, liver, cortex, and striatum samples (see Supplementary Material for Methods).

**FIGURE 2:**
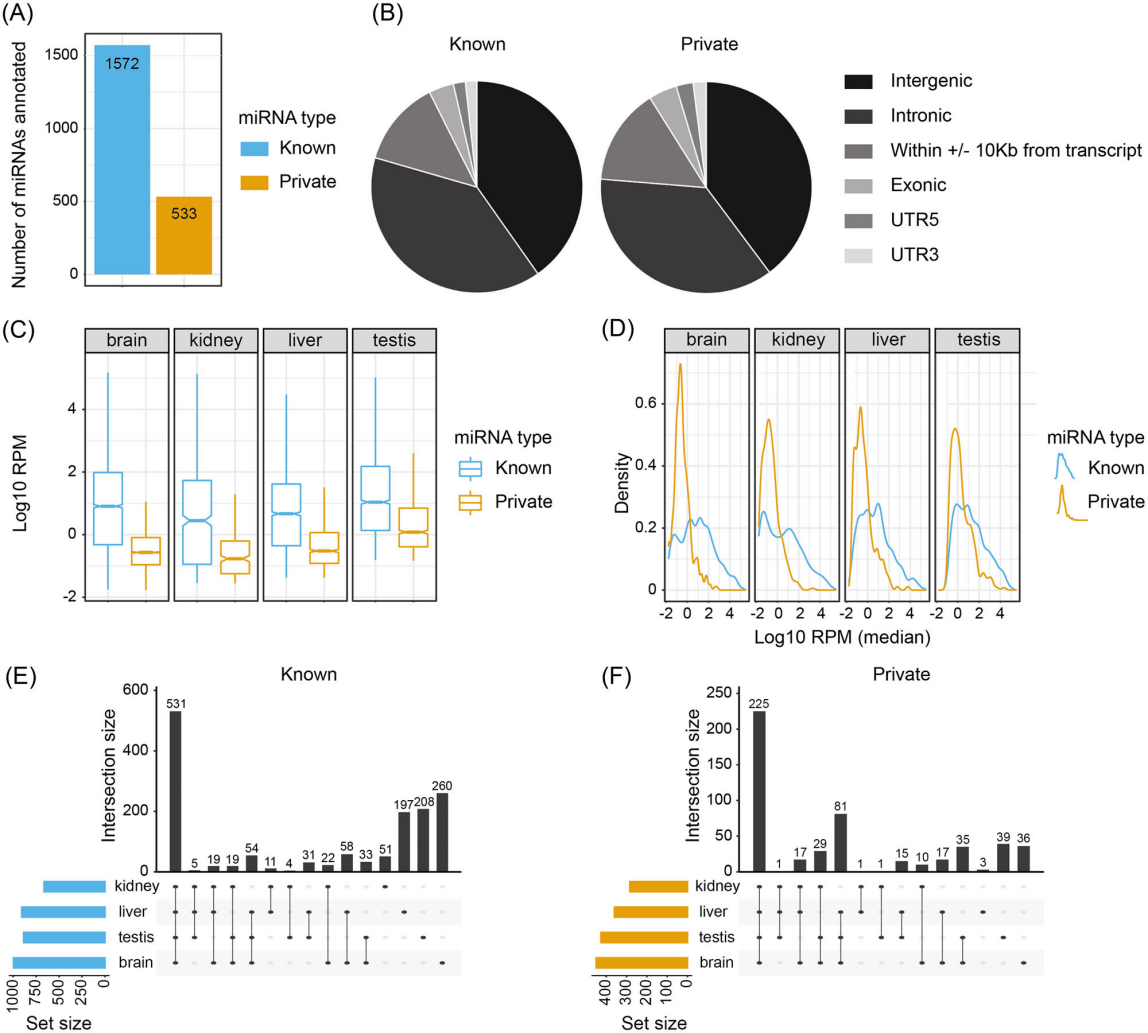
Annotation of miRNAs in the *P. discolor* genome. (A) In total, 2105 miRNAs were identified, of which 1572 were known miRNAs and 533 were private. (B) Genomic location of known and private miRNAs. The vast majority in both categories were encoded within intergenic and intronic regions. (C,D) Expression of miRNAs in the brain (cortex + striatum), liver, kidney, and testes from five adult *P. discolor* bats displayed as (C) Log^10^ reads per million (RPM) represented as box plots or (D) density plots. The horizontal lines in the box plots indicate the median expression of miR‐337‐3p, boxes extend between the first and third quartile, while whiskers extend by 1.5 times the interquartile range as per the default setting in R. In general, known miRNAs are more highly expressed and have more miRNAs in the high expression range than private miRNAs. (E,F) UpSet plots demonstrate the tissue‐specific expression pattern of (E) known and (F) private miRNAs. The vast majority of miRNAs are expressed in all four tissues tested. Known miRNAs also had large numbers of tissue‐specific miRNAs in the liver, testis, and brain.

This led to the identification of 2105 miRNAs in the *P. discolor* genome (Figure 2), a large increase from the 335 known miRNAs previously identified.^40^ One thousand five hundred and seventy‐two of these miRNAs were known miRNAs already identified in another genome, as described in miRbase v22.^47^ Five hundred and thirty‐three were not present in miRbase yet were determined from their sequence properties to be likely to encode a miRNA using miRDeep2^48^ and thus termed “private” miRNAs. This private class may represent miRNAs that have newly emerged in *P. discolor* or in Chiroptera—although their presence or absence in other bat species is yet to be determined. The vast majority of miRNAs were located in intergenic regions and introns (∼80%), but miRNAs were also encoded within 10 Kb around the transcriptional start and end sites, and in exons, 5’UTRs and 3’UTRS (Figure 2B and Table S3). This pattern is comparable for known and private miRNAs and similar to that found in other mammals.^49^

The newly generated small RNA‐Seq data also allowed us to assess the expression profile of these miRNAs across tissues. We observed that in all tissues assessed, the known miRNAs tended to be more highly expressed than the private miRNAs (Figure 2C). This is consistent with the previously reported hypothesis that newly emerged miRNAs tend to have very low expression levels, which gradually increase over evolutionary time.^50^ However, a small number of private miRNAs show very high expression values (Figure 2D), which would predict a strong effect of these highly expressed miRNAs on the targets they regulate. This may point to a selective advantage provided by the function of these miRNAs in *P. discolor*. When we examined the expression of miRNAs across different tissues, the patterns were comparable for both known and private miRNAs (Figure 2E,F). We observed that the brain had the greatest number of miRNAs expressed, both known and private, commensurate with the transcriptomic complexity of this tissue. The majority of miRNAs were expressed in multiple tissues, with only a small proportion of miRNAs restricted to one specific tissue. Those miRNAs that were tissue‐specific were most likely to be found in the brain and testes (known or private miRNAs) or liver (known miRNAs only).

Using functional data to annotate 3’UTR regions is crucial given their difficulty to accurately predict from purely sequence data, the large number of possible isoforms, and the spatiotemporal variability of 3’UTR isoform usage. Accurate maps of 3’UTR regions make it possible to predict how miRNAs and RNA binding proteins will interact with 3’UTR sequences to affect stability, localization, and protein expression. We applied MACE sequencing to the same testes, liver, cortex, and striatum samples from five individuals to survey the 3’UTR usage in these bats. We were able to map 24,133 3’UTRs across all tissues with an average length of ∼1.8 Kb and a median length of 886 nt (Table 2). In all tissues, about half of the 3’UTRs identified (∼11 K) matched with the previous annotations,^40^ while a large proportion of 3’UTRs was novel (∼5.5 K) or extended in length (∼7.5 K) compared to the previously published annotations (Table 2).

**TABLE 2:** 3’UTR regions identified using MACE sequencing for testes, liver, and brains of *P. discolor* bats

Tissue	Total #	# matching previous annotations	# with increased UTR length	# novel	Median length
All	24,133	11,162	7467	5504	886
Liver	21,280	10,203	6553	4524	938
Testis	22,678	10,700	6884	5094	791
Brain	23,216	10,479	7623	5114	1055

### Part I: Conclusions and future directions

Herein, we report annotations for coding genes and noncoding regulatory elements (miRNAs and the 3’UTRs that are targeted by them) that represent a substantial improvement over previous bat genomes—including our prior *P. discolor* annotations.^40^ We increased the number of coding genes and transcripts overall, and brought the BUSCO score to 99%, suggesting that very few gene models are now missing from the annotation. There was a marked increase in the annotation of 5’ and 3’UTRs and noncoding miRNAs, which greatly increases our ability to understand regulatory mechanisms in this species. The role of miRNAs in refining transcriptomic profiles is known to be important for brain development and neural circuit activity and we anticipate that miRNA‐facilitated regulation of expression will also be important for the development and functioning of circuitry involved in vocal learning. Our new annotations give the possibility to explore the regulatory networks driven by microRNAs underlying this and other complex phenotypes to an extent not possible before. These improved coding and noncoding annotations will facilitate future studies into gene–function relationships using both candidate gene or omics‐level approaches and will enhance our ability to find evolutionary relationships between genes, regulatory elements, and phenotypes both within *P. discolor* and across species.

### Part II: Neurobiology

Successful vocal learning requires a range of abilities, including auditory perception and processing, vocal motor control, template matching, learning, and memory, all of which are supported by complex neural circuitry.^9^, ^51^, ^52^ While detailed circuit maps have been generated in vocal learning birds like zebra finches,^53^ little is currently known about the specific neural circuitry that underlies vocal learning in mammals. Bats have a long history of neuroethological research, and while bats have been classified as vocal learners for more than 20 years,^4^ neurobiological investigations related to bat vocal behavior have largely focused on echolocation (see the Introduction for references). These studies have led to a much greater understanding of the regions of the brain and neural circuitry involved in auditory perception, processing, and navigation. Coupled with the broadly conserved mammalian structure of the brain, there is an ideal baseline for intensive studies into mammalian vocal learning in bats. In *P. discolor*, it is crucial that we understand the basic brain morphology of the species and develop the tools necessary to build on this understanding to define specific circuitry underlying complex vocal learning behavior. To this end, we have been developing multiple complementary approaches in *P. discolor*, including electrophysiology, neuroimaging, connectivity tracing, and histology. Since electrophysiology is routinely employed in bats, we refer to the primary papers rather than presenting such data herein (e.g., Refs. 54, 55, 56, 57, 58). Instead, we focus here on our recent neuroimaging, tracing, and genetic mapping approaches. These, together with controlled behavioral assays in this species, will be key to revealing the neural basis of vocal learning in bats.

#### Magnetic resonance imaging

Given the important role that structure plays in the functional capabilities of a brain, it is essential to have a thorough understanding of the structural organization of an organism's brain. In *P. discolor*, meticulous histological approaches have given a broad view of brain structure as well as detailed maps of primarily subcortical components of the brain.^59^ In the cortex, electrophysiological studies have given a deep understanding of the location and computational properties of auditory cortical regions.^28^, ^32^, ^55^ Missing, however, were ways to observe the whole brain structure and activity, map brain‐wide connectivity, and measure brain‐wide changes in the organization following interventions. Neuroimaging approaches give an effective way to address these gaps.

**FIGURE 3:**
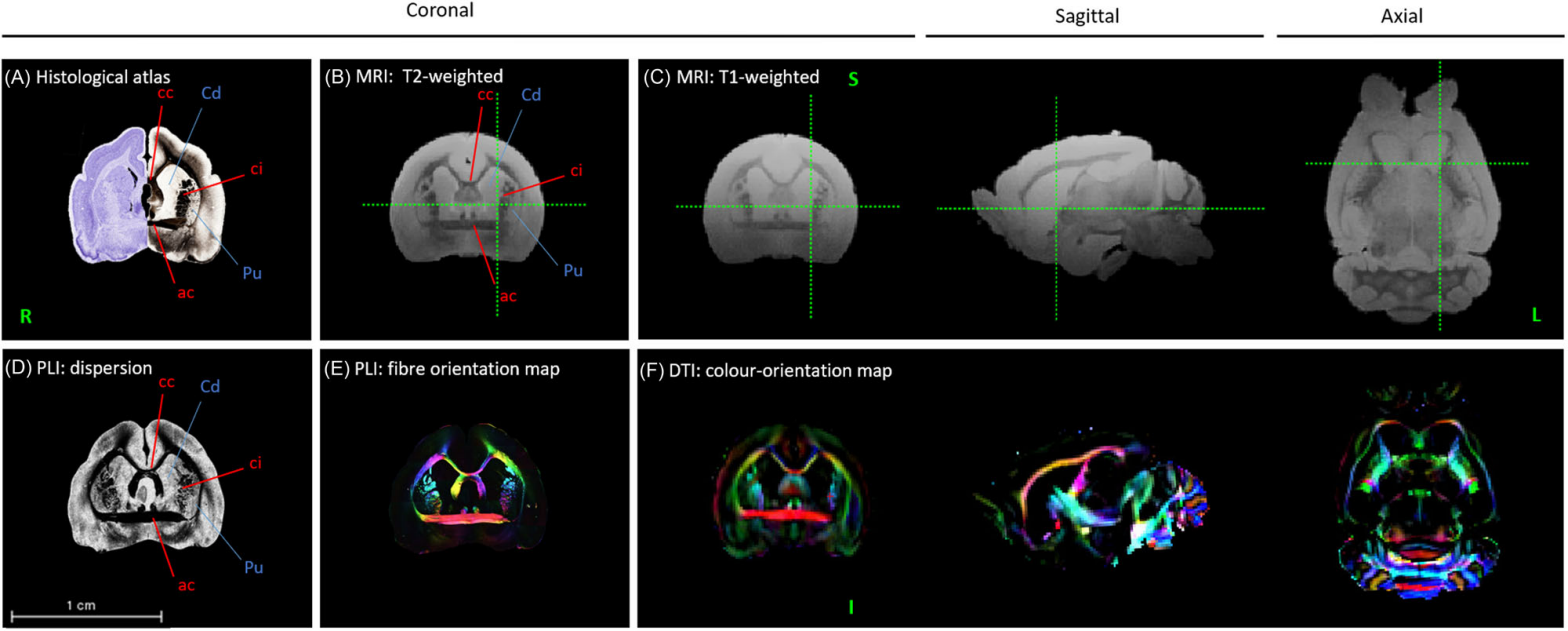
Neuroimaging data provide anatomical information of the *P. discolor* brain. (A) An adapted plate of a previously published histological coronal *P. discolor* brain atlas. Modified from atlas plate 16 (combined Nissl and acetylcholine stain), published in Ref. 59. (B) Coronal T2‐weighted MR image of a female adult *P. discolor* bat brain (voxel size 0.1 × 0.1 × 0.1 mm). (C) Coronal, sagittal, and axial T1‐weighted MR images of the same brain (voxel size 0.1 × 0.1 × 0.1 mm). (D) PLI dispersion image of a matching coronal slice of the same brain. (E) PLI fiber orientation map of a matching coronal slice of the same brain. (F) Coronal, sagittal, and axial color orientation maps based on diffusion tensor imaging (DTI) of the same brain (voxel size 0.15 × 0.15 × 0.15 mm). Red annotations indicate white matter structures and blue annotations indicate gray matter structures. Green crosshairs refer to the same location across the different viewing planes and indicate slice depth. Abbreviations: ac, anterior commissure; cc, corpus callosum; Cd, caudate nucleus; ci, capsula interna; Pu, putamen. S, I, R, and L refer to the following orientations: S, superior; I, inferior; R, right side of the brain; L, left side of the brain. The scale bar represents 1 cm.

We have begun to utilize magnetic resonance imaging (MRI) and specifically diffusion tensor imaging (DTI)^60^ to investigate the macro‐scale structure and connectivity of the *P. discolor* brain. Additionally, we are employing polarized light imaging (PLI)^61^ on the same brains to investigate the meso‐scale connectivity of these brains. In Figure 3, we present initial MRI, DTI, and PLI data of an adult *P. discolor* brain. We report both T1‐ and T2‐weighted MRI images (see Table S4 for scanning conditions) and fractional anisotropy (FA) color direction mapping based on DTI, as well as dispersion and fiber orientation maps based on PLI. By comparing the coronal sections of the MRI, DTI, and PLI images with precise histological maps from the published atlas,^59^ we observed strong concordance and were able to identify the same gray matter structures (e.g., the caudate nucleus and putamen; Figure 3A, in blue) and white matter structures (e.g., the corpus callosum, anterior commissure, and capsula interna; Figure 3A, in red) in our structural MR images (Figure 3B,C), PLI images (Figure 3D,E), and diffusion data of the brain (Figure 3F). These data show the feasibility and accuracy of neuroimaging‐based approaches in *P. discolor* bats.

#### Tracing auditory pathways

**FIGURE 4:**
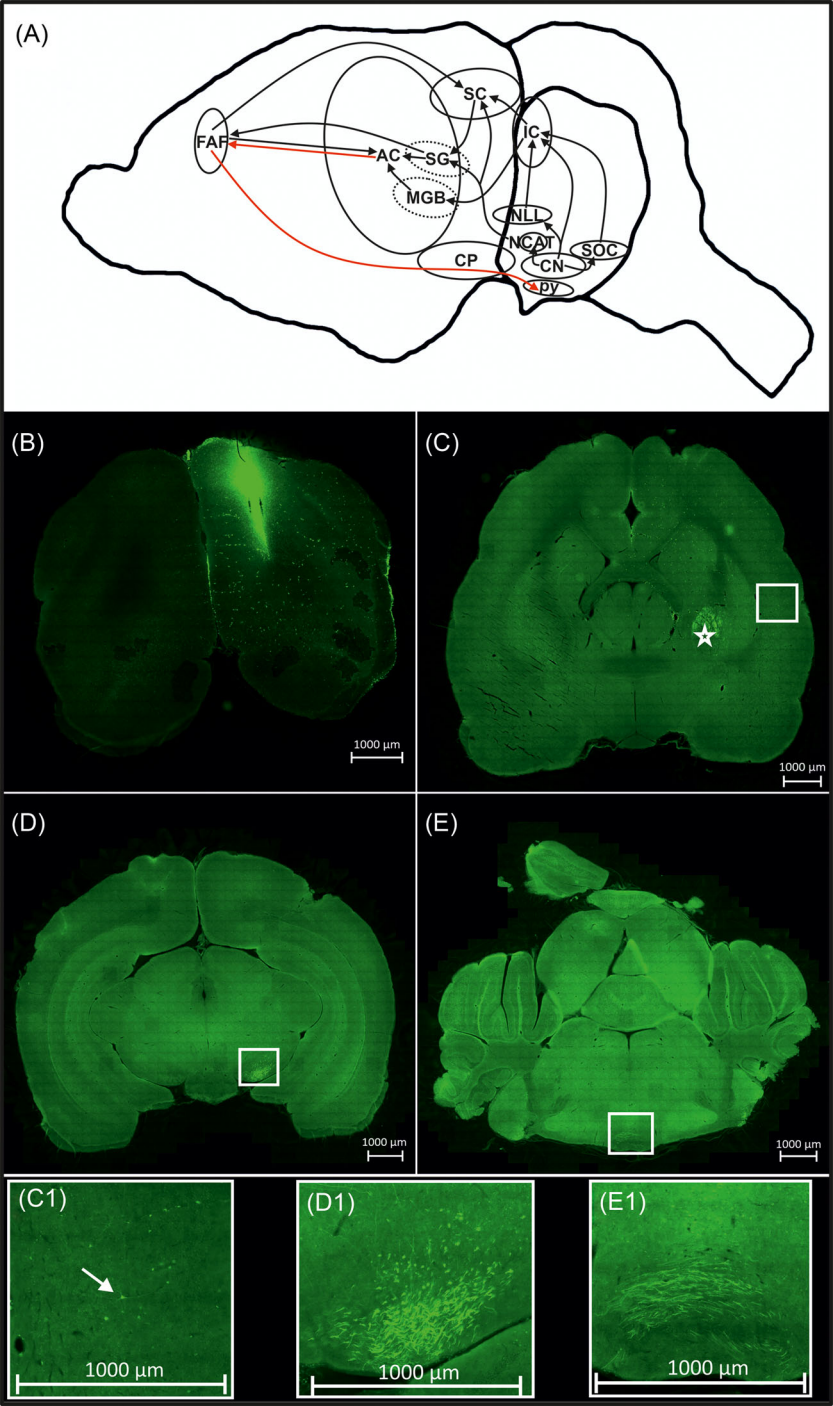
Auditory inputs into the frontal auditory field (FAF) and its descending projections to a possible motor pathway. (A) Summary diagram of ascending auditory pathways to the cortex in the bat and projections from a frontal cortical region to a possible motor pathway. Auditory input via the VIII nerve (not shown) enters the cochlear nucleus (CN). The extralemniscal pathway consists of the NCAT (nucleus of the central acoustic tract), followed by either the suprageniculate body (SG) and/or the superior colliculus (SC). Projections from the SG connect to either the auditory cortex (AC) or the frontal acoustic field (FAF). Alternatively, auditory information can be relayed from the CN to the IC (inferior colliculus) either via the SOC (superior olive complex), NLL (nucleus of the lateral lemniscus), or via direct projection. The IC projects either to the SC or the MGB (medial geniculate body), which itself projects to the AC. The AC projects to the FAF while also receiving FAF projections. Finally, the FAF projects to both the SC and the pyramidal tract (py) via the cerebral peduncle (CP). Arrows in black are known connections in either *P. discolor* or closely related species, and red connections are based on evidence from this study. Diagram based on work by Pollak and Casseday.^65^ (B) The injection site of the tracer into the frontal cortex (Plates #7 and 8 in the reference atlas^59^). (C) Labeled neurons (see arrow in highlighted insert C1) in AC and strong labeling of fibers (star) in the capsula interna (Plate #17). (D) Strong labeling of fibers (see highlighted insert D1) in the cerebral peduncle (Plate #26). (E) Strong labeling of fibers (see highlighted insert E1) in the pyramidal tract, note switching of hemispheres (Plate #34 in the reference atlas^59^).

Auditory perception and processing are important components of vocal learning. Thanks to the extensive use of bats to study the neuroethology of echolocation, there is a good understanding of auditory pathways in the bat brain. The ascending auditory pathway in bats is broadly conserved with that of other mammals,^62^, ^63^ with auditory input ascending from the cochlear nucleus either via the extralemniscal pathway (CN→NCAT→SG/ CN→NCAT→SC, see Figure 4A and legend for abbreviations) or via the inferior colliculus (IC), directly or indirectly (CN→IC/ CN→SOC→IC/ CN→NLL→IC). These pathways then input onto the auditory cortex (AC) via the auditory thalamus (SG/MGB). Evidence from several bat species has implicated another specialized frontal cortex region in the auditory processing pathway known as the frontal auditory field (FAF), which has been suggested to be a homolog of the mammalian PFC/M2. This brain region receives auditory input directly from the AC^62^, ^63^ and also from the SG via the extralemniscal pathway. Since the FAF responds to acoustic stimuli and has strong projections to the superior colliculus (SC), it has been hypothesized to be involved in sensorimotor integration, sound evaluation, and implementation of auditory‐related behaviors (e.g., attention, ear movements, etc.).^63^ These properties suggest the FAF may represent a region of interest for bat vocal learning circuitry and the perception and processing of vocal signals.

Given these properties of the FAF and the importance of understanding auditory processing pathways more generally for future vocal learning studies, we sought to trace the connectivity of the FAF region with other parts of the auditory pathway in *P. discolor*. Injecting a dextran tracer into the FAF (Figure 4B) showed clear connectivity to the AC (Figure 4C) in line with previous evidence of bidirectional connectivity of these cortical regions.^62^, ^63^ In addition, we observed strong labeling in fibers from the FAF down through the capsula interna of the striatum (Figure 4C, star), through the cerebral peduncle (Figure 4D, no axonal terminals were observed suggesting passing through rather than connection to), and finally into the pyramidal tract (Figure 4E). Proof of the pyramidal tract lies in the crossover. The majority of stained fibers switch from the ipsilateral to the contralateral side. The connectivity to the pyramidal tract was unexpected as it was not found previously, and it suggests a role for the FAF in implementing the motor activity. In mice, similar connectivity to the pyramidal tract was observed when tracers were injected into the secondary motor cortex.^64^ These data suggest the possibility that bats may have developed a specialized auditory premotor/motor cortical area, and we hypothesize that the FAF could act as a sensorimotor integration point for auditory processing and vocal production/initiation.

#### Genetic markers of brain regions

Using gene expression patterns to define brain regions within species and for comparative exploration of brain properties across species is an approach that is widely used in other systems, such as humans, mice, and birds.^66^, ^67^, ^68^, ^69^, ^70^, ^71^ Conserved expression patterns are not proof of shared functionality and further approaches, such as electrophysiology, must be employed to determine function. However, the extensive mapping performed in other species makes it a powerful first step in exploring the properties of brain regions and their potential convergence across diverse vocal learning species. This has been illustrated in zebra finches, where expression patterns within song circuitry are well defined, and genes that delineate specific brain regions or show differential expression during vocal behaviors have been identified.^69^, ^70^, ^72^, ^73^, ^74^ Comparative work has drawn parallels between the expression patterns in zebra finch song circuitry with human brain regions involved in speech, showing some convergent expression patterns and potentially convergent functionality.^75^, ^76^, ^77^ Here, we explored the *P. discolor* brain using a histological approach to observe gross structures and expression patterns of selected genes.

**FIGURE 5:**
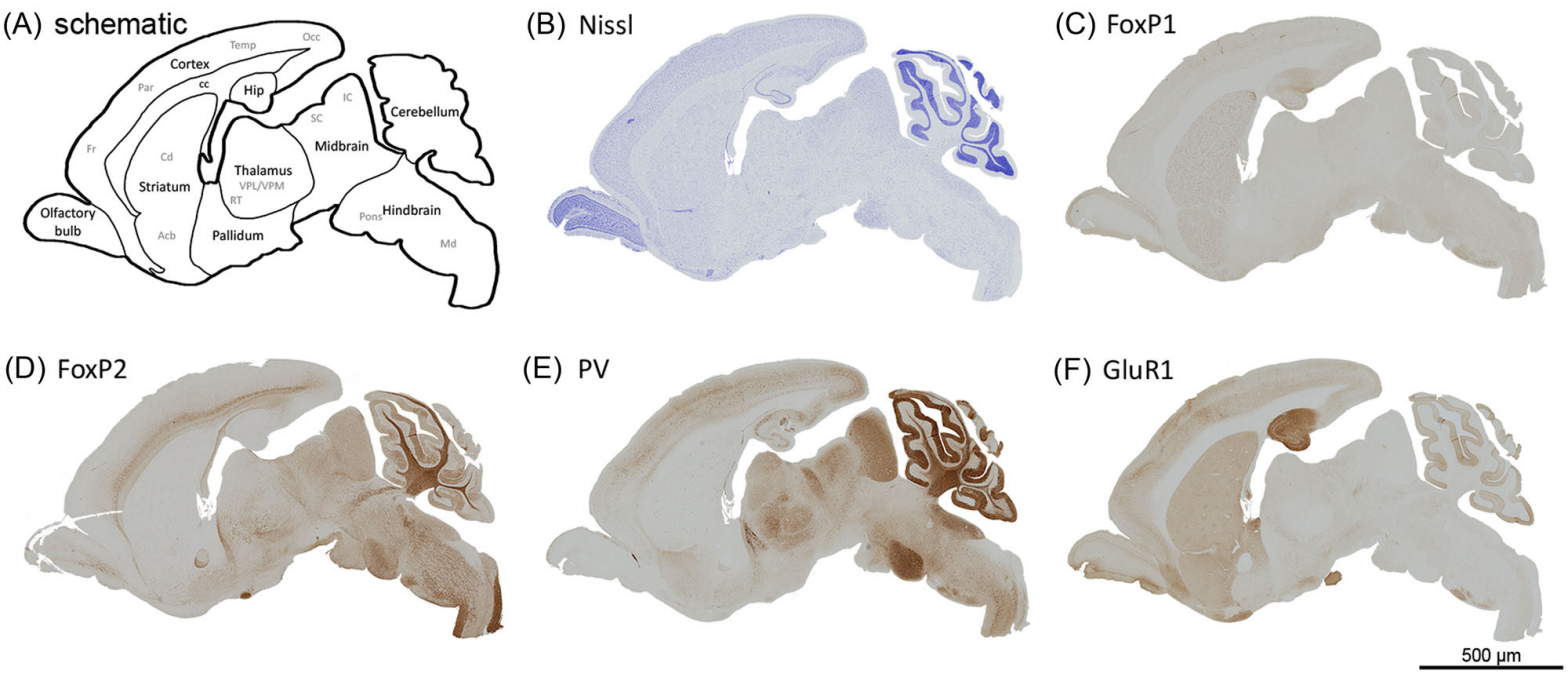
Sagittal view of the adult *P. discolor* bat brain via histology. (A) Schematic representation indicates key anatomical structures used for protein expression analysis. The main anatomical structures are written in black, while subregions marked out by protein expression are in gray. (B) Nissl stain of the sagittal slice. Immunohistochemical detection of protein expression of (C) FoxP1, (D) FoxP2, (E) parvalbumin, and (F) GluR1. Foxp2 N16 antibody produces high levels of fiber staining that can be seen in panel D in the corpus collosum and in the white matter of the cerebellum and brainstem. For a clearer representation of the staining, the image brightness of all the stained images was altered using a linear adjustment of the mid‐tones of the levels parameter in Photoshop (mid‐tones of Image of Nissl stains were adjusted to 0.5, and images of protein expression were adjusted to 0.3). The scale bar indicates 500 μm. Abbreviations: Acb, nucleus accumbens; cc, corpus callosum; Cd, caudate nucleus; Fr, frontal cortex; Hip, hippocampus; IC, inferior colliculus; Occ, occipital cortex; Md, medulla; Par, parietal cortex; RT, reticular thalamic nucleus; SC, superior colliculus; Temp, temporal cortex; VPL, ventral posterolateral thalamic nucleus; VPM, ventral posteromedial thalamic nucleus.

We generated a sagittal view of the *P. discolor* brain (Figure 5A) as this allowed us to identify a range of cortical and subcortical structures within a single slice. Regions were identified based on Nissl staining (Figure 5B) with the help of a published coronal brain atlas.^59^ As observed previously from the existing coronal atlases,^59^, ^78^ the *P. discolor* brain shows parallels to the overall structure of the mouse brain. While stains such as Nissl are useful to identify the overall brain structures, the expression patterns of specific genes can help to refine structural maps and identify potential convergence or divergence across species. We explored the gross expression patterns of four genes that have been previously implicated in a speech in humans or vocal learning in songbirds (see Table S5 for staining conditions). We chose the FOXP1 and FOXP2 transcription factors since disruptions of either gene in humans lead to speech and communication‐related deficits.^5^, ^79^ These genes have subsequently been implicated in zebra finch song circuitry and learning.^80^, ^81^, ^82^, ^83^ Parvalbumin (PV) encodes a calcium‐ion binding protein and was chosen as it is enriched in motor neurons used for speech production in humans (versus macaques). PV is also a marker of the song motor pathway in zebra finches^84^ and is differentially expressed in the human laryngeal motor cortex and the songbird ortholog.^75^ These findings suggest a potential convergent role for PV in brain circuitry involved in speech and vocal learning.^84^ Lastly, the Glutamate receptor 1 gene (GluR1, also known as GRIA1) is an excitatory glutamatergic neurotransmitter (AMPA) receptor that was chosen as it is differentially expressed in the song circuitry of zebra finches.^72^

Another benefit of the sagittal view is that we can explore the length of the cortex from anterior to posterior in a single slice. In the *P. discolor* cortex, FoxP1 (Figure 5C), PV (Figure 5E), and GluR1 (Figure 5F) were expressed widely, but very little FoxP2 (Figure 5D) could be observed. These data are consistent with our previous findings for FoxP1 and FoxP2 in coronal maps.^85^ In songbirds, FoxP2 shows very little expression in regions of the song circuitry that are thought to be homologous to the mammalian cortex, such as LMAN (lateral portion of the magnocellular nucleus of the anterior neostriatum), HVC (high vocal center), and RA (robust nucleus of the arcopallium) .^86^, ^87^ By contrast, in rodents, Foxp2 is strongly expressed in deep layers of the cortex during development and into adulthood.^88^, ^89^ Thus, cortical FoxP2 expression may be more similar between songbirds and the *P. discolor* bat compared to more closely related rodents.

In the *P. discolor* cortex, PV and Glur1 demonstrated broadly inverse gradients of expression. PV tended to be weakest in anterior cortical regions and became stronger in posterior cortical regions (Figure 5E), while Glur1 was strongest in anterior cortical regions and weaker in posterior cortical regions (Figure 5F). However, both proteins showed some regional variability. In the striatum, FoxP1 was widely expressed, while Foxp2, PV, and Glur1 were more sparsely expressed. Glur1 could be seen to mark out striosomes, which is consistent with primate and mouse staining for this protein.^90^, ^91^, ^92^ All four proteins were present in the thalamus, but each demonstrated a specific pattern of expression that marked out different combinations of thalamic nuclei. Interestingly, PV and Glur1 showed an inverse pattern of expression in the somatosensory thalamus (VPL/VPM nuclei) where PV expression was enriched compared to surrounding tissue, while Glur1 expression was reduced compared to surrounding tissue. In the hippocampus, Glur1 is strongly expressed, PV and Foxp1 were moderately expressed, and Foxp2 was absent. In the cerebellum, Foxp1 is not expressed. Purkinje cells showed the expression of PV and Foxp2, while the granular layer was marked out by PV and Glur1. The IC, SC, and pons were all strongly marked out by PV and moderately marked out by Foxp2. Expression of Glur1 and Foxp1 was notably absent in these three structures.

Overall, the patterns observed for FOXP2, FOXP1, PV, and GluR1 in the *P. discolor* brain were broadly consistent with mice and other mammals (with the noted exception of cortical FoxP2 expression). We expect any differences in expression related to vocal learning would be very subtle and that these changes would be found in specific populations of neurons rather than being large brain‐wide changes in expression. This likely would be due to the importance of these genes for general brain development and function. Indeed, this is what has been observed in songbird mapping studies where specific nuclei of the song circuitry show subtle changes in these genes. Work is now underway to perform a detailed comparison of the expression of these genes across bats, birds, rodents, and humans to uncover any convergent expression or patterns that are shared by vocal learning species.

### Part II: Conclusions and future directions

We have built upon the strong history of neuroethological research in bats to expand the tools that can be applied in *P. discolor* so that future work can exploit them to address the neurobiology of vocal learning in bats. What is striking from all the approaches used is that the *P. discolor* brain shares strong structural homology with rodent brains. Albeit a much larger brain (about 1.5 times the size of a lab mouse brain), the gross structures of the *P. discolor* brain are easily identifiable when compared with rodents, which is highly beneficial for undertaking comparative work. There are, however, clear differences between species. For example, the AC in *P. discolor* (and in echolocating bats more generally) is greatly expanded compared to the mouse, and the FAF may be a specialized sensorimotor integration point for auditory processing and vocal‐motor production. Future work will apply the approaches discussed herein to explore vocal learning circuitry in the *P. discolor* brain and uncover the more subtle differences that may be present when comparing to rodents or other species.

Neuroimaging approaches have been used in bats in a few prior studies,^93^, ^94^ but this represents the first published study in *P. discolor*. Comparing our data with published histological atlas allowed us to validate the MR imaging approach and demonstrate its accuracy in revealing brain structures. Our findings will now make it possible to use neuroimaging to perform brain‐wide connectivity studies and unravel the *P. discolor* connectome. Such MR imaging and related connectivity analyses have limitations, such as relatively low resolution, and in the case of connectivity analyses, modeling the structure of the brain's connectivity indirectly by measuring the water density/diffusion rather than directly measuring it. By combining these data with complementary methods, such as PLI, which is a direct measure of connectivity with enhanced resolution, we can validate MR‐based data with histological data to circumvent these downsides.

In the future, targeted probabilistic tract‐tracing studies from brain areas involved in vocal communication in the *P. discolor* brain will enable direct comparisons between vocal learning in bats and speech in humans—where there is a wealth of DTI data publicly available^95^, ^96^, ^97^, ^98^, ^99^, ^100^, ^101^, ^102^, ^103^—something typically not possible when employing other invasive tracing techniques. MR imaging techniques will also allow the assessment of brain‐wide quantitative differences in developmental stages or sexes. To date, we have not observed major differences between the sexes; however, there are some social calls that are predominantly used between mothers and pups. Future work will explore whether, like in some other species, there are sex‐specific differences in vocal learning. In the future, imaging techniques will also allow exploration of whole brain effects following experimental interventions, such as during learning paradigms or following genetic manipulations (see Part III). Work is also underway to develop functional neuroimaging approaches *in vivo* to explore the whole brain activation pattern during vocal behaviors in *P. discolor*.

Our tracing studies in *P. discolor* together with previous studies in other bat species^63^ show the feasibility of this technique in bats. The work presented herein sheds light on a brain region involved in bat auditory processing specifically the FAF. This is a region identified specifically in bats,^62^, ^63^, ^104^ and its relationship with other mammalian brain regions is not yet clear but has been hypothesized to have homologies to the mammalian PFC.^105^, ^106^ We confirmed a connection between the FAF and the AC, corroborating auditory inputs to the FAF from the AC in *P. discolor*. Strikingly, we uncovered a novel projection of the pyramidal tracts pointing to possible involvement of the FAF in motor functions. This involvement has been speculated in the past as previous studies found strong projections from the FAF into the SC.^63^ These projections led to the conjecture that the FAF may be involved in sensory–motor integration,^63^ which would be consistent with our current findings. Given the involvement of the FAF in auditory processing, it is intriguing to consider that this may be related to auditory–vocal–motor integration, but a combination of tracing, electrophysiological recoding, and stimulation studies is required to test this hypothesis.

The histological data presented herein demonstrate the feasibility of applying similar genetic mapping approaches in bats to those used in songbirds^107^, ^108^, ^109^ to explore potential homologies with human brain areas. Although antibodies are not routinely raised toward bat proteins, the high‐quality annotations we produced for the *P. discolor* genome (Part I) mean that in most cases, the conservation of epitopes targeted by existing antibodies can be used to predict the specificity of an antibody for detecting bat proteins. Despite the ∼65 million years of evolution since the divergence of the bat lineage,^36^ the protein‐coding regions of bat genes remain sufficiently conserved such that the majority of the antibodies we tested that were generated for use in rodents or humans were able to be applied successfully to the bat brain. Combining multiple methods will allow us to discover the neural circuitry underlying bat vocal learning in a targeted and brain‐wide manner and discover any homologies with birds or humans. These combined methods can include: coupling mapping and transcriptomic approaches to elucidate expression patterns of specific regions, structural approaches, such as MRI and tracing, and functional neurophysiological approaches to define the activity of these regions.

### Part III: Genetic manipulations in bats

Observing the natural state of a behaving animal can reveal potential mechanisms underlying that behavior. An effective way to demonstrate causal links between neurogenetic mechanisms and behavior is to perturb gene function. After genetic manipulations, effects on molecular pathways, brain development, and behavior can be measured.

In widely used model organisms like flies or rodents, the creation of a germline transgenic animal to reveal causal mechanisms has become routine thanks to their ease and speed of generation.^110^, ^111^ However, even with recent advances in CRISPR‐Cas9 genome editing,^112^ the creation of germline transgenics in bats is challenging given the low number of offspring that results from their uniparous reproduction, which generally only takes place once or twice per year. Alternate approaches like transient transgenics are a valuable way to flexibly alter gene expression that avoids many challenges associated with generating germline transgenics.

Herein, we describe our efforts to manipulate *FoxP2* expression in the brain of living *P. discolor* bats to facilitate future exploration of its role in vocal learning behavior and associated neural circuitry. This was of particular interest given the identification of a role for FOXP2 in speech and language in humans and for vocal learning in zebra finch songbirds^51^, ^113^—presumably via a convergently evolved mechanism. In songbirds, it has been shown that both reducing (via shRNAs‐mediated knockdown^6^, ^114^) and increasing (by providing the full‐length protein via viral constructs^7^) the expression of FoxP2 could disrupt vocal learning abilities, demonstrating the importance of correct dosage of FoxP2 for this behavior. Manipulating FoxP2 in bats presents the first opportunity to explore the role of this speech‐related gene in a vocal learning mammal, and thus we sought to generate viral vectors for this purpose and show their efficacy in the brain.

**FIGURE 6:**
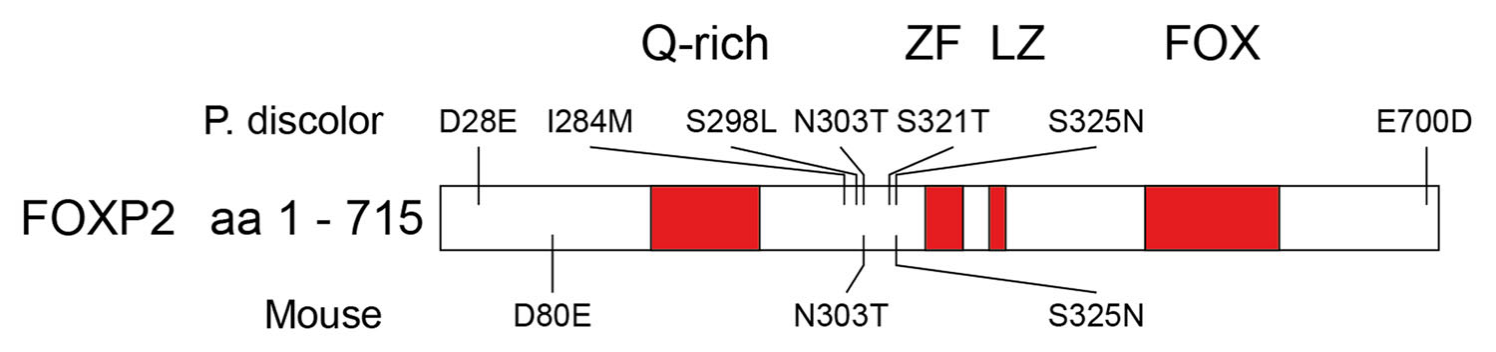
Representation of the human FOXP2 protein displaying known functional domains (in red) and amino acid differences found in *P. discolor* bats (above) and in mice (below). No changes are found in the functional domains of the protein. Some further variability is detected in the low complexity Q‐rich tract as shown in File S2 (clustal alignment), although this is hard to resolve. Abbreviations: FOX, forkhead‐box DNA binding domain; LZ, leucine zipper domain; Q‐rich, glutamine‐rich region; ZF, zinc finger domain.

Bats represent a powerful model to study the role of genes involved in vocal learning and human speech and language.^8^ To exploit this, we must first understand any sequence changes that have taken place over the evolution of these genes. *FoxP2* is one of the most highly conserved genes between mice and humans.^115^ Excluding minor differences within the low complexity Q‐rich tract, only three amino acid changes separate the protein‐coding sequence of these species.^115^ This high level of conservation is broadly maintained over mammals (Figure S1). Our annotations show that *P. discolor* Foxp2 protein is also highly conserved (Figure 6) displaying only seven amino acid differences with the human protein, excluding the polyQ region (Figure S2). The forkhead‐box (FOX) DNA binding domain that characterizes this protein is 100% conserved. This suggests that the functionality of FoxP2 and the target genes that it regulates are highly conserved across *P. discolor* bats and humans. This conservation suggests that these bats can act as model systems to interrogate the functional role of genes like FoxP2 in learned vocal behavior.

#### Increasing FoxP2 expression in living bats

**FIGURE 7:**
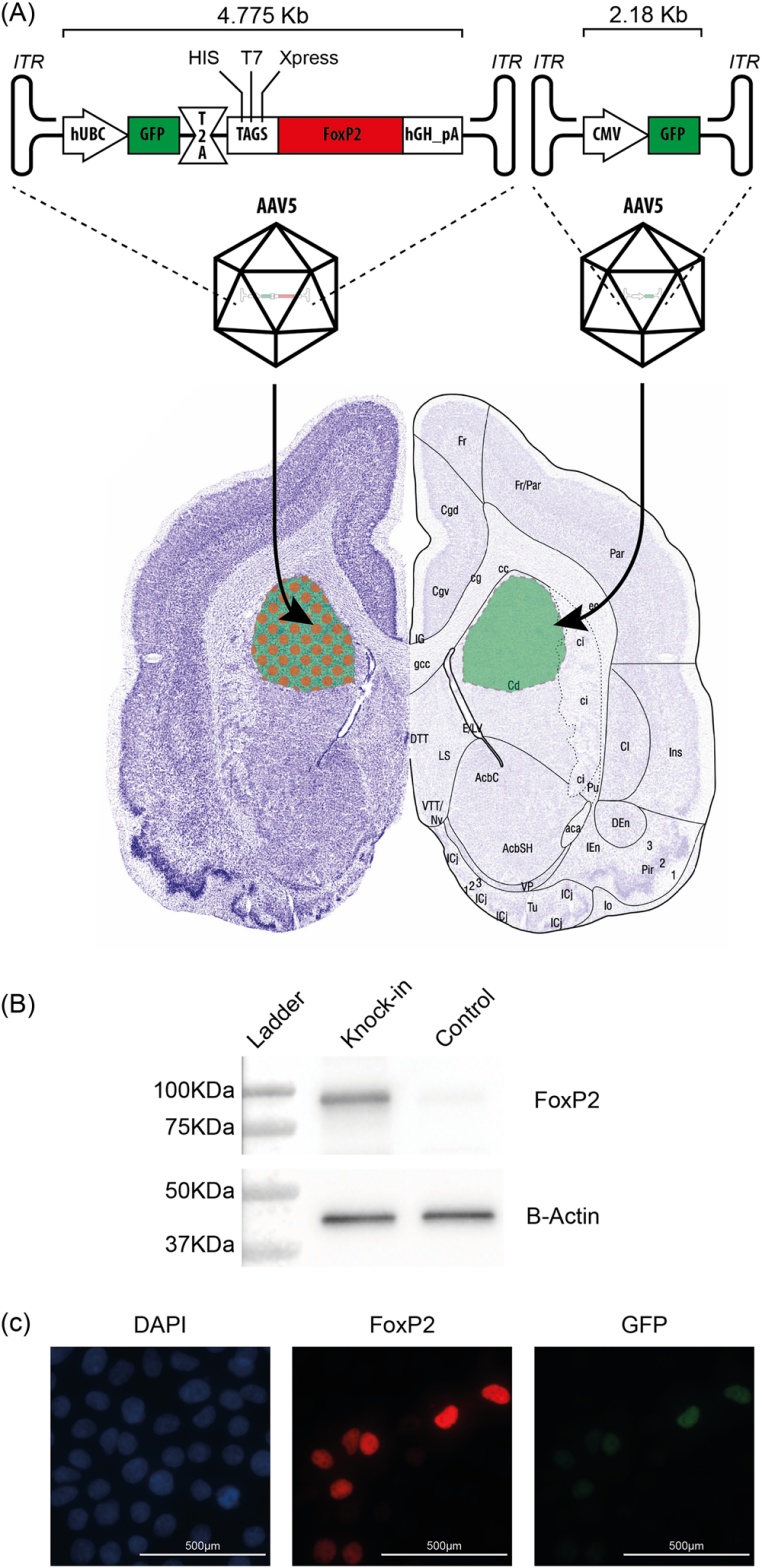
Design and *in vitro* testing of *FOXP2* transgenic constructs. (A) Schematic of the expression construct used to overexpress FoxP2. We expressed the *P. discolor* FoxP2 under the control of the human ubiquitin promoter (hUBC). To facilitate detection, we fused FoxP2 with a series of small peptidic tags (HIS‐tag, FLAG‐tag, T7, and Xpress®), and we used a T2A system to separately express the FoxP2 protein from the same promoter driving GFP expression. The GFP protein acted as a marker of the region of infection in the brain. To stabilize the transcript and enhance expression, we used the termination and polyadenylation signal of the human growth hormone (hGH). A premade virus coding for GFP under the control of a CMV promoter was purchased from Virovek (Hayward, California) (AAV5‐CMV‐GFP) to be used as a control. (B) *In‐vitro* testing of the expression cassette. We transfected HEK293T/17 cells (ATCC, CRL‐11268) with the FoxP2 expression cassette from panel (A) and detected strong overexpression of FoxP2 via western blot compared to untransfected HEK293T/17 cells. The full membrane image is shown in Figure S2. (C) Subcellular localization of the ectopically expressed FoxP2. We transfected HEK293T/17 cells (left panel, DAPI stain) with the FoxP2 expression cassette from panel (A) and detected strong overexpression of FoxP2 (middle panel) in IF using a FoxP2 antibody. GFP (right panel) indicates the presence of transgenic rather than endogenous protein in these cells. FoxP2 expression in the nucleus shows the expected localization of the transgenic protein.

To study the function of FoxP2 in bat neurobiology and behavior, we set out to overexpress FoxP2 in the brain of a living bat. We created a construct that would express the *P. discolor* FoxP2 alongside a GFP marker protein under a shared promoter. This was packaged into an AAV5 virus for delivery into the brain (Figure 7A). This design used the T2A system^116^ to express two separate proteins from the same transcript to allow the inclusion of a fluorescent marker within the small size limits of AAV packaging. The GFP marker allows localization of the infected area without the need for a tagged fusion FoxP2 protein. This was important as a large tag may interfere with the molecular functions of FOXP2 by possibly hindering physical interactions between protein and DNA. We initially tested the efficacy of this construct *in vitro* in HEK293 cells and confirmed that it expressed the full‐length FoxP2 protein (Figure 7B) and that, as expected, the protein was localized to the nucleus of cells (Figure 7C).

**FIGURE 8:**
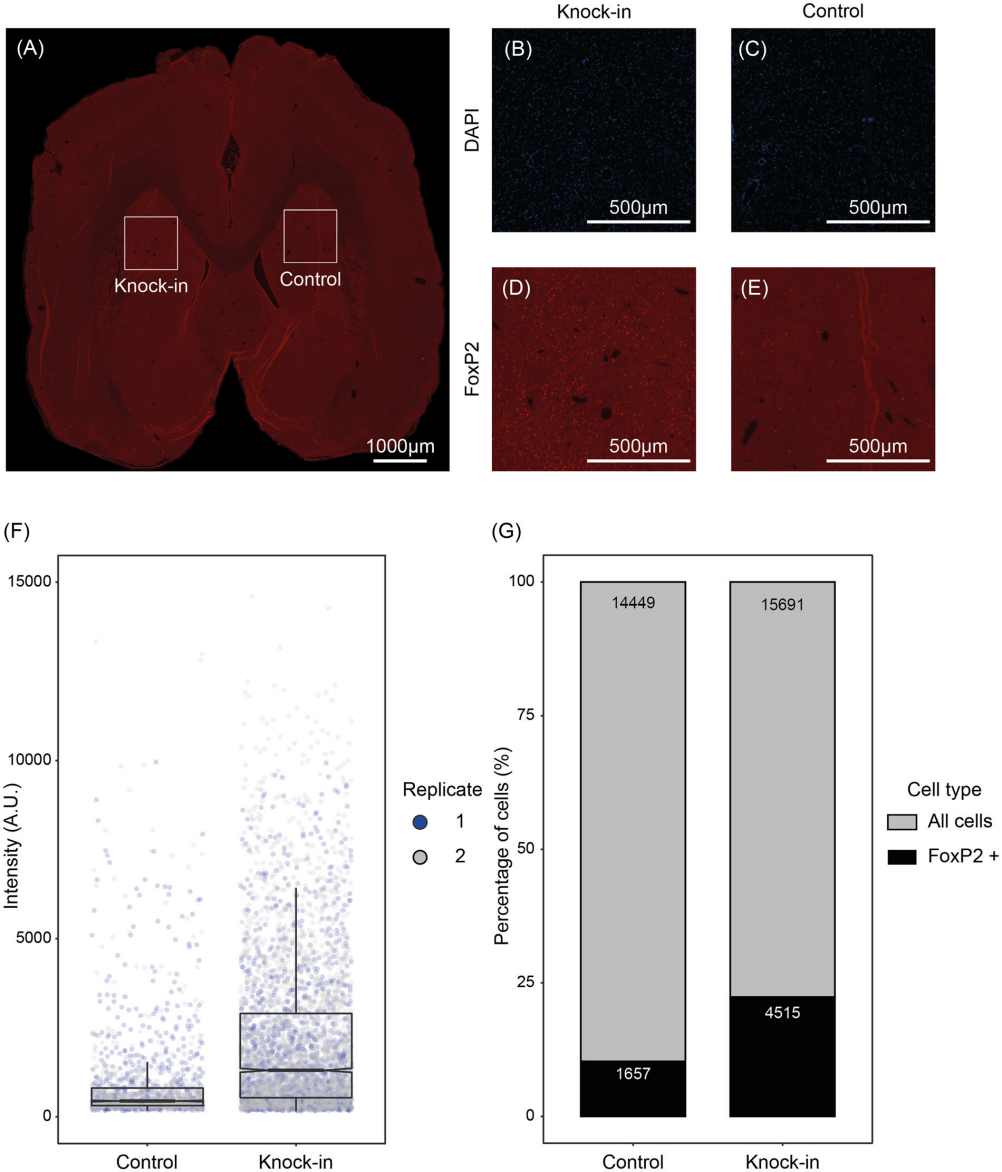
*In vivo* validation of transient transgenic bats. (A) IF of the *P discolor* brain 10 days after the injection of the viral constructs shows overexpression of FoxP2 in the left hemisphere (injection with the UBC‐GFP‐FoxP2 AAV5 virus) compared to the right hemisphere (injection with the control CMV‐GFP AAV5 virus). (B,C) Zoom in of the injected area. DAPI staining indicates cellular integrity in both hemispheres. (D,E) Strong overexpression of FoxP2 in the left hemisphere was detected with an antibody against FoxP2 (MABE415; Table S5). (F) To quantify the overexpression of FoxP2, we measured the intensity of the signal in IF using MetaMorph (Molecular Devices). The knockin hemisphere showed an approx. three‐fold increase in FoxP2 median expression compared to the control hemisphere (*t‐*test, *p* < 2.2e‐16). (G) Following infection with the UBC‐GFP‐FoxP2 AAV5 cassette, we recorded an increase in the number of cells positive for FoxP2 in the knockin hemisphere compared to the AAV5‐CMV‐GFP control hemisphere. Overall, 4515 cells (of 15,691 total cells detected in the region) were found to express FoxP2 in the knockin hemisphere compared to 1657 in the control hemisphere (of 14,449 total cells detected in the region). (F,G) Represent the combined data from two separate brain slices analyzed in the same way (see Table S6 for individual values).

We injected the AAV5‐GFP‐FoxP2 virus into the striatum of adult bats, in one hemisphere only (Figures 7A and 8A). Into the other hemisphere, we injected the control AAV5 carrying only a GFP reporter (AAV5‐CMV‐GFP) using the equivalent coordinates to allow within‐individual comparisons (Figures 7A and 8A). After 10 days of incubation, we assessed the expression of FoxP2 via immunofluorescence, which clearly showed that the virus had infected striatal neurons (with a spread of approx. 1500 μm medial‐lateral and 900 μm dorsal‐ventral; Figure 8B–E). The overall median intensity of the FoxP2 signal was almost tripled from 443 to 1305 (Figure 8F and Table S6), and the number of FoxP2‐positive neurons in this region had significantly increased from ∼1600 to ∼4500 (Figure 8G and Table S6). This clearly shows the efficacy of the delivery method and the successful transgenic overexpression of FoxP2 in a living bat brain.

### Part III: Conclusions and future directions

This is the first successful manipulation of gene expression in a living bat and the first generation of a transgenic vocal learning mammal. These data clearly show a successful manipulation of bat striatal neurons *in vivo* to overexpress FoxP2. The short incubation time (10 days) was only intended as proof of principle and future work will explore the influence of manipulating FoxP2 expression on molecular pathways, cell morphology, neural circuitry, and vocal learning behavior after longer incubation times. This will give a chance for the changes that occur downstream of FoxP2, molecular or neural, to exert their influence on phenotypes using the toolkit we describe herein (Parts I and II). We will also extend these studies to the consequences of FoxP2 overexpression when introduced into other regions of the brain. For example, one benefit of the overexpression construct is that we can increase expression where FoxP2 is present but sparse (e.g., the adult striatum), or in places where there is little FoxP2 expression (e.g., the adult cortex).^78^ We have also been developing knockdown constructs to reduce FoxP2 expression, and these will be applied to relevant brain regions where expression is high (e.g., juvenile AC) to observe consequences on brain development and behavior. The first genetic manipulations in a vocal learning animal were in the zebra finch and demonstrated that tight control of FoxP2 expression in the striatum is essential for normal vocal learning abilities.^6^ In a part of the adult striatum (area X in birds), loss of FoxP2 expression disrupted song circuitry and behavior in zebra finches.^6^ In juvenile zebra finch area X, both underexpression and overexpression of FoxP2 caused some similar perturbations to vocal learning behavior and neuromolecular pathways.^6^, ^7^ Our FoxP2 overexpression model will allow us for the first time to make direct comparisons between these pioneering bird studies and a vocal learning mammal.

The ability to manipulate gene expression *in vivo* in the bat brain will allow a major step forward in understanding neurogenetic contributions to vocal learning. It will allow future exploration of not only *FoxP2*, but other coding genes, noncoding RNAs, and molecular pathways in this complex behavior. These include those implicated in songbird studies to determine if these are evolutionarily conserved mechanisms, as well as those from human studies—particularly genes implicated in speech/language disorders. This would reveal both fundamental mammalian mechanisms involved in complex communication, as well as mechanisms by which these genetic changes can cause disorders in children. As each new gene is addressed, it will be important to tailor the transgenic design and target the appropriate brain regions. For example, cross‐species comparisons could introduce the expression of a gene into a region where it is found in birds, but not bats. Studies based on clinical genetics could knock down gene expression to recapitulate the effects of the patient mutation. Building a toolkit of techniques and utilizing the newest technologies to generate these transgenics will provide maximal flexibility and greatly advance our ability to understand the neurogenetic mechanisms underlying mammalian vocal learning in bats and over evolution.

## DISCUSSION

We have outlined approaches in the *P. discolor* bat to explore the neural and genetic mechanisms underlying vocal learning. Many of these approaches are routinely applied in other animal systems like mice or songbirds but have not been utilized in this species before. If we are to harness the potential of bats to reveal the biological and evolutionary mechanisms of vocal learning, such tool development is crucial. Moreover, it is important that we do this in an integrative manner, linking the different levels of investigation from genes to brains to behavior. While we have addressed a few key areas from our current work, ongoing and future work aims to expand these tools through their exploration of techniques such as fMRI, monosynaptic and viral tracing, *in vivo* calcium imaging, and optogenetics. The tractability of *P. discolor* makes this an ideal bat species in which to develop and test these methods. However, we do not want to imply that studies should only focus on this species. We hope that any advances we make in tool development in *P. discolor* could be propagated and employed in other vocal learning bat species. As noted in the Introduction, several bat species have been identified with vocal learning abilities and importantly, the behaviors displayed by these bats vary greatly. Thus, to gain a true understanding of bat vocal learning, interrogation of bats across the family tree is crucial. Furthermore, studies in diverse bat species, and potential comparisons with other mammalian and bird vocal learners, are necessary to gain an evolutionary perspective on vocal learning.

## AUTHOR CONTRIBUTIONS

S.C.V. conceived and designed the experiments, contributed to data analysis and interpretation, and wrote the manuscript. P.D. and S.G.H. contributed to the conception and design of experiments and acquired and analyzed/interpreted data in Parts I/III and II/III, respectively. I.A.vT. contributed to the design of experiments and acquired and analyzed/interpreted data in Part II. N.H., J.M., and M.W. contributed to the design of experiments and acquired and analyzed/interpreted data in Part II. U.F. and P.H. contributed to the design of experiments and analyzed data in Part II. K.L. contributed to the design of experiments and analyzed data in Part I. M.H. and A.E.M. contributed to the data collection and analysis in Part I. G.M.H. contributed to the data collection and analysis in Part III. All authors contributed to the writing and editing of the manuscript and authorized the final version of the manuscript.

## COMPETING INTERESTS

The authors declare no competing interests.

### PEER REVIEW

The peer review history for this article is available at: https://publons.com/publon/10.1111/nyas.14884.

## Supporting information

Figure S1. Mammalian species tree showing the diversity of the FOXP2 protein sequence across 48 mammals. Branches represent the number of differing amino acid sites relative to the Homo sapiens sequence. Each vertical line represents one amino acid change in the species. Species with some unknown residues are represented with “*”
Figure S2. *In vitro* testing of FOXP2 transgenic constructs. Complete western blot image corresponding to Figure 7 of the main text.
Table S1. Tissues used for transcriptomic studies.
Table S2 List of species used for the annotation of known miRNAs.
Table S3. Genomic locations of known and private miRNAs.
Table S4. MR scanning conditions.
Table S5. Antibodies used for immunostaining (IHC and IF).
Table S6. Quantification of FoxP2 upregulation in bat striatum––individual replicate values.
Materials and Methods

File S1. *P. discolor* updated annotations.

File S2. FoxP2 Clustal Protein Alignment

## Footnotes

a According to convention, human *FOXP2* is denoted in uppercase, mouse *Foxp2* is in lowercase, and all other species have *FoxP2* in mixed upper and lowercase.

b The current metric for reference genomes is summarized as 6.7.P5.Q40.C90, where the notation is *x*.*y*.*P*.*Q*.*C*: *x* = log_10_[contig NG50], *y* = log_10_[scaffold NG50], *P* = log_10_[haplotype phase block NG50], *Q* = QV base accuracy, and *C* = percentage of the assembly assigned to chromosomes. However, this is likely to develop rapidly in line given the speed of technological and method development in this area.
