# Supplementary material for: The pale spear‐nosed bat: A neuromolecular and transgenic model for vocal learning
Source: Ann N Y Acad Sci. 2022 Sep 7;1517(1):125–42. doi: 10.1111/nyas.14884 (PMC9826251; doi:10.1111/nyas.14884)
Supplement: Supplementary file 1 — Figure S1. Mammalian species tree showing the diversity of the FOXP2 protein sequence across 48 mammals. Branches represent the number of differing amino acid sites relative to the Homo sapiens sequence. Each vertical line represents one amino acid change in the species. Species with some unknown residues are represented with “*” Figure S2. In vitro testing of FOXP2 transgenic constructs. Complete western blot image corresponding to Figure 7 of the main text. Table S1. Tissues used for transcriptomic studies. Table S2 List of species used for the annotation of known miRNAs. Table S3. Genomic locations of known and private miRNAs. Table S4. MR scanning conditions. Table S5. Antibodies used for immunostaining (IHC and IF). Table S6. Quantification of FoxP2 upregulation in bat striatum––individual replicate values. Materials and Methods [file NYAS-1517-125-s002.docx]

**SUPPLEMENTARY MATERIALS**

**Table of contents**

Supplementary Files Page 1

Supplementary Figures Page 2

Supplementary Tables Page 4

Materials and Methods Page 7

**SUPPLEMENTARY FILES**

File S1. *P.discolor* updated annotations

(file name: nyas14884-sup-0002-SupMat.bed)

File S2. FoxP2 Clustal Protein Alignment

(file name: nyas14884-sup-0003-SupMat.clustalnum)

**SUPPLEMENTARY FIGURES**

**Figure S1.**


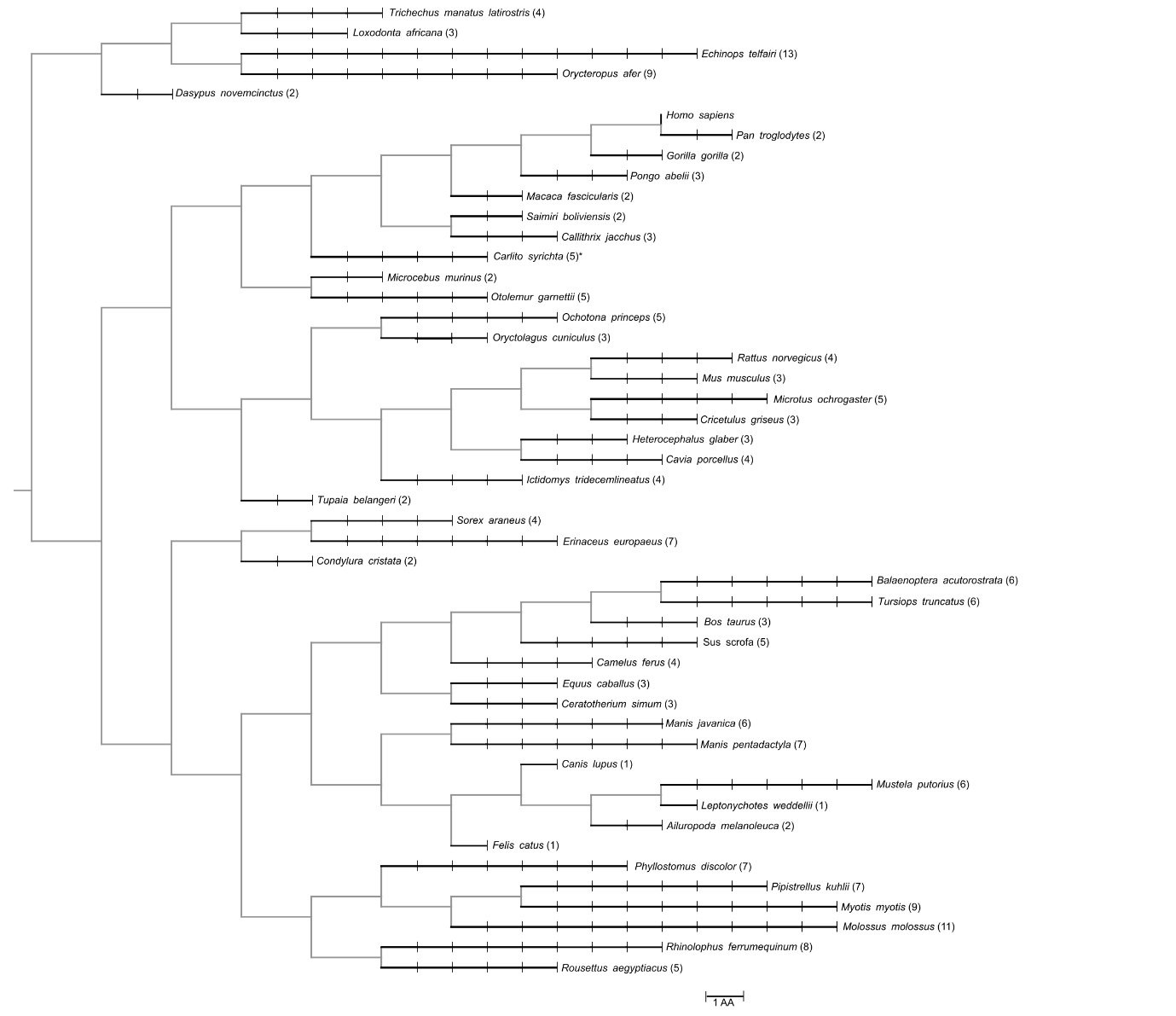


***Figure S1. Mammalian species tree showing the diversity of the FOXP2 protein sequence across 48 mammals.*** *Branches represent the number of differing amino acid sites relative to the Homo sapiens sequence for the 715 amino acids of Isoform I, excluding the Poly-Q region (639 aa total). Each vertical line represents one amino acid change in the species (total number per species is given in brackets). Species with some unknown residues are represented with ‘*’.*


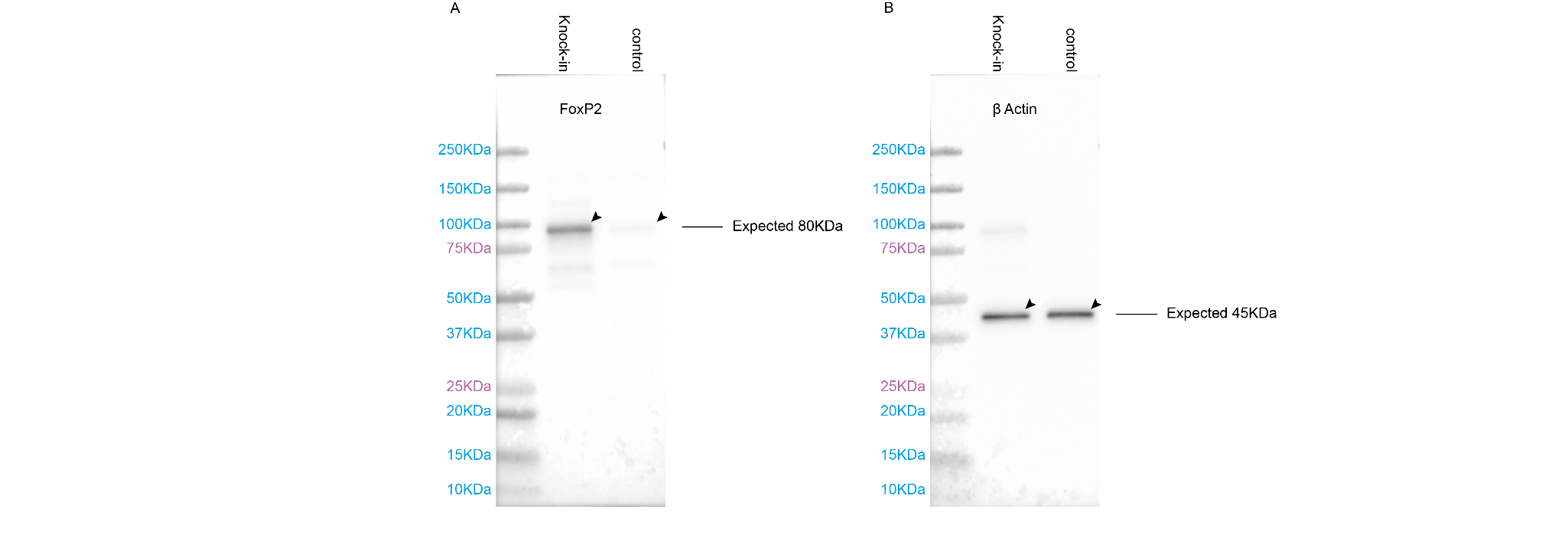


***Figure S2. In vitro testing of FOXP2 transgenic constructs.*** *Complete western blot image corresponding to Figure 7 of the main text.*

**SUPPLEMENTARY TABLES**

**Table S1. Tissues used for transcriptomic studies**

|  |  |  |  | **Sequencing technology** | | |
| --- | --- | --- | --- | --- | --- | --- |
| **Species** | **Animal number ID** | **Tissue** | **Brain Hemisphere** | **MACE** | **smallRNA** | **mRNA** |
| *P. discolor* | 2 | Frontal cortex | Right | x | x | x |
| *P. discolor* | 3 | Frontal cortex | Right | x | x | x |
| *P. discolor* | 4 | Frontal cortex | Right | x | x | x |
| *P. discolor* | 5 | Frontal cortex | Right | x | x | x |
| *P. discolor* | 7 | Frontal cortex | Right | x | x | x |
| *P. discolor* | 2 | Striatum | Right | x | x | x |
| *P. discolor* | 3 | Striatum | Right | x | x | x |
| *P. discolor* | 4 | Striatum | Right | x | x | x |
| *P. discolor* | 5 | Striatum | Right | x | x | x |
| *P. discolor* | 7 | Striatum | Right | x | x | x |
| *P. discolor* | 2 | Liver |  | x | x | x |
| *P. discolor* | 3 | Liver |  | x | x | x |
| *P. discolor* | 4 | Liver |  | x | x | x |
| *P. discolor* | 5 | Liver |  | x | x | x |
| *P. discolor* | 7 | Liver |  | x | x | x |
| *P. discolor* | 2 | Testis |  | x | x | x |
| *P. discolor* | 3 | Testis |  | x | x | x |
| *P. discolor* | 4 | Testis |  | x | x | x |
| *P. discolor* | 5 | Testis |  | x | x | x |
| *P. discolor* | 7 | Testis |  | x | x | x |
| *P. discolor* | 3 | Cerebellum |  |  |  | x |
| *P. discolor* | 3 | Arm muscle |  |  |  | x |
| *P. discolor* | 3 | Lung |  |  |  | x |
| *P. discolor* | 3 | Blood |  |  |  | x |
| *P. discolor* | 3 | Heart |  |  |  | x |
| *P. discolor* | N/A | Kidney |  |  | x | x |
| *P. discolor* | N/A | Wingclip |  |  |  | x |
| *P. discolor* | N/A | Primary fibroblasts |  |  |  | x |

**Table S2 – List of species used for the annotation of known miRNAs**

| **Species abbreviation (miRBase)** | **Species** |
| --- | --- |
| hsa | Homo sapiens |
| pal | Pteropus alecto |
| dme | Drosophila melanogaster |
| bta | Bos taurus |
| cel | Caenorhabditis elegans |
| ssc | Sus scrofa |
| rno | Rattus norvegicus |
| efu | Eptesicus fuscus |
| mmu | Mus musculus |
| ocu | Oryctolagus cuniculus |
| gga | Gallus gallus |
| tgu | Taeniopygia guttata |
| eca | Ecquus caballus |
| mml | Macaca mulatta |
| cja | Callithrix jacchus |
| mdo | Monodelphis domestica |
| ppy | Pongo pygmaeus |
| ptr | Pan troglodites |

**Table S3. Genomic locations of known and private miRNAs**

|  | **Known miRNAs** | | **Private miRNAs** | |  |
| --- | --- | --- | --- | --- | --- |
| **Genomic compartment** | **Number of miRNAs** | **Percentage** | **Number of miRNAs** | **Percentage** |  |
|  |  |  |  |  |  |
| Intergenic | 1494 | 40,2% | 311 | 36,5% |  |
|  |  |  |  |  |  |
| Introns | 1456 | 39,2% | 339 | 39,8% |  |
|  |  |  |  |  |  |
| 10Kb around transcript | 488 | 13,1% | 126 | 14,8% |  |
|  |  |  |  |  |  |
| CDS_Exons | 141 | 3,8% | 37 | 4,3% |  |
|  |  |  |  |  |  |
| 5'UTR_Exons | 68 | 1,8% | 22 | 2,6% |  |
|  |  |  |  |  |  |
| 3'UTR_Exons | 65 | 1,8% | 17 | 2,0% |  |
|  |  |  |  |  |  |

**Table S4. MR scanning conditions**

| Anatomical scan | Repetition time | Echo time | Flip angle | FOV | resolution | matrix | b-value | directions |
| --- | --- | --- | --- | --- | --- | --- | --- | --- |
| T1 | 20 ms | 3.88 ms | 15 degrees | 25.6x25.6x25.6 mm | 0.1x0.1x0.1 mm | 256x256x256 | - | - |
| T2 | 1800 ms | 33 ms | 90 degrees | 15x15x22 mm | 0.1x0.1x0.1 mm | 150x150x220 | - | - |
| DTI | 500 ms | 30.22 ms | 90 degrees | 14.4x14.4x24 mm | 0.15x0.15x0.15 mm | 96x96x160x69 | B=3000 | 64 dir |

**Table S5. Antibodies used for immuno-staining (IHC & IF)**

**Table S6. Quantification of FoxP2 upregulation in bat striatum – individual replicate values***.*

| **Condition** | **Replicate** | **Median Intensity** | **Average Intensity** | **SD Intensity** | **Counts** |
| --- | --- | --- | --- | --- | --- |
| CONTROL | 1 | 511 | 1131 | 1489 | 635 |
| KNOCK IN | 1 | 1117 | 1871 | 1969 | 1521 |
| CONTROL | 2 | 415 | 806 | 1677 | 1004 |
| KNOCK IN | 2 | 1418 | 2282 | 2388 | 2845 |

**Materials and Methods**

**Animals**

The *P. discolor* bats originated from a breeding colony situated in the Department Biology II of the Ludwig-Maximilian University of Munich. Animals were kept under semi-natural conditions (12 h day/12 h night cycle, 65%–70% relative humidity, 28°C) with access to food and water *ad libitum*. Animals undergoing any surgical procedures were kept physically separated from other animals. All the experiments complied with the principles of laboratory animal care and the regulations of the current version of the German Law on Animal Protection. The number of animals used in terminal experiments was reported to the Munich veterinary office.

**PART I**

**Transcriptomics sample collection and RNA sequencing**

Animals were euthanized by an intraperitoneally applied lethal dose of pentobarbital (0.16 mg/g bodyweight). Tissues were dissected within 10 minutes of euthanasia and snap-frozen in liquid nitrogen. According to German Law on Animal Protection a special ethical approval is not needed for this procedure, but the number of sacrificed animals was reported to the district veterinary office. All tissues were lysed Qiazol (cat #79306) and RNA was extracted and sequenced as previously described (Jebb et al). Total RNA extraction and purification was conducted using the QIAGEN RNeasy kit (Cat. No. 74104) to collect two separate fractions from each tissue containing RNA (>200nt long) and small RNAs (< 200nt long), respectively. For the large RNA fraction (> 200nt) the quality and quantity of RNA was assessed via Bioanalyzer or Tapestation before library preparation and sequencing, performed by the commercial service (Novogene or BGI). Sequencing was performed on Illumina NovaSeq platform, with 150nt long paired-end reads at a minimum depth of 30M reads. For the small RNA fraction, the total amount of RNA was quantified using a Nanodrop and the presence of a peak at approx. 20nt was assessed using a Bioanalyzer by BGI prior to library prep and sequencing. Standard library with the addition of UMI (Unique Molecular Identifier) was generated by BGI and sequencing was performed using DNBSEQ™ technology platforms at minimum 20M reads depth. An overview of tissues and RNA samples is available in Supplementary Table 1.

**MACE sequencing and 3’UTR mapping**

The 3'UTRs of *P. discolor* species were experimentally identified via MACE sequencing^1^, which allows precise mapping of transcribed poly(A) sites. A minimum of 200ng of the large RNA fraction from each tissue (Supplementary Table 1) was used for the MACE library preparation and sequencing at GenXPro GmbH (Frankfurt Main, Germany). Sequencing was performed on Illumina Hiseq2000 or NextSeq500 platforms at 20 M Reads depth; read length 75bp 8bp of which will be dedicated to the TrueQuant Adapter (Unique Molecule Identifiers). Data analysis was performed following the pipeline published in [PMID: 29656876] (with minor modifications) to obtain the set of candidate poly(A) clusters indicating the end of 3’UTR for each gene. All details and the relevant scripts can be found at <https://github.com/aksenia/utr3ome>. Since the original PASSannotate script only annotates the poly(A) sites with respect to the nearest gene locus, we refined the annotations to assign the candidate poly(A) sites to transcripts within each gene locus. The resulting MACE and single-exon Iso-seq 3’ UTR datasets were merged into a non-redundant set of single-exon 3’ UTRs (the majority), which were used for the downstream analyses.

**Annotation pipeline**

Briefly, IsoSeq data was first analyzed as in Jebb et al.^2^ to produce high-quality ORF predictions. Then, we implemented a strategy to classify and filter transcripts-based features such as known canonical splice sites, known non-canonical splice sites, novel canonical-splice sites, and novel non-canonical splices. A small set of transcripts, with suboptimal features were not used as input for the gene annotation. For example, fusion transcripts (chimeras that include more than one gene), intra-priming (transcripts with more than 85% or at least 10 contiguous adenines within 20 bp upstream of the 3’ end), low coverage (transcripts supporting coding regions by less than 3 reads), RT-switching predictions (an exon-skipping pattern due to a retrotranscription gap caused by secondary structures in expressed transcripts), nonsense-mediated decay (premature stop codons), and intron retention were features all identified as suboptimal. However, when possible, some of these transcripts were used to annotate UTRs. Transcript features used for classification were identified using SQANTI3 (PMC5848618) and TAMA-GO (PMC7218625). Then, new TOGA predictions were generated using an updated version (Kirilenko et al. In Review; <https://github.com/hillerlab/TOGA> version 1.0). We used as reference genomes human (hg38), mouse (mm10), and the greater mouse-eared bat (*Myotis myotis*). Finally, additional RNAseq data from tissues were generated and analyzed, as described in Ref. 2. Protein alignments also used as annotations were not modified from those generated in Ref. 2. Evidences (RNAseq transcripts, reclassified IsoSeq transcripts, TOGA predictions, and proteins) were integrated using EVM (PMC2395244), and downstream steps to annotate UTRs, enrich the annotation with non-coding RNAs, and assign gene names were performed as described in Ref. 2.

**microRNA annotation**

We developed a multi-step procedure to annotate microRNAs starting with small RNA sequencing data and based on the workflow previously published as part of the miRanalyzer tool^3^. We first downloaded the full list of mature microRNA sequences and IDs from miRBase v22 and then parsed it to include only the entries from the most closely related and well-annotated mammalian species. The procedure is split into two parts, in the first part known miRNAs are identified using Bowtie by mapping the reads derived from small RNA sequencing experiments to the mature sequences downloaded from miRBase. In the second part, any reads that do not align to known miRBase entries are used as input to miRDeep2 in order to identify putative “private” microRNAs that are then ranked in miRDeep2 based on the likelihood of being a real microRNA. The mapping to miRBase v22 entries is done in 4 substeps, with different parameters. After each step, the unmapped reads are used as input for the next step. In the first two substeps the reads are mapped to the human (hsa-) and mouse (mmu-) mature miRNAs and then to the precursor sequences, respectively. Due to the large evolutionary distance between these species and P. discolor, the following parameters were used in Bowtie: -l 18 -v 3 -k 3 --best --strata –norc. The remaining reads were then mapped to the miRNAs annotated for the only two bat species in miRBAse, first to Eptesicus fuscus (efu-) then to Pteropus alecto (pal-). Given the closer evolutionary distance between these species and P. discolor, the following parameters were used in Bowtie: -l 18 -v 1 -k 3 --best --strata –norc. The remaining reads were then mapped to all other mammalian species (see Supp Table 2) using the following parameters: -l 18 -v 3 -k 3 --best --strata –norc. This procedure allowed us to identify within the expressed small RNAs the sequences that resemble more closely known, real microRNAs in other species. This resulted in 1572 known microRNAs that were named using the numbering from the existing database entry but changing the prefix to “pdi-”. Manual curation was necessary to remove identical sequences assigned to miRNAs annotated in different species. Any reads that did not map to the microRNAs known in the species above, were used as input to the miRDeep2 algorithm, which resulted in 533 “private” microRNAs (called “novel” in the miRDeep2 package). The working list of miRNAs, their sequences and the genomic locations will be made available upon request, and it will be submitted to miRBase (https://www.mirbase.org/) as part of an upcoming dedicated publication.

**PART II**

**Magnetic Resonance Imaging (MRI) and Polarized Light Imaging (PLI)**

1 adult female *P. discolor* bat was sedated with MMF (medetomidine (Dorbene®, Zoetis), midazolam (Dormicum®, Hoffmann-La Roche) and fentanyl (Fentadon®, Albrecht) at a dosage of 0.4, 4.0 and 0.04 µg/g body weight, respectively), euthanised with an intraperitoneal injection of pentobarbital (Narcore®, Boehringer Ingelheim Vetmedica GmbH, 0.16 mg/g bodyweight) and perfused transcardially by injecting approximately 200 ml of phosphate-buffered saline (PBS; pH 7.4) with 0.9% NaCl and Liquemin (with pH of 7.4) for 10 minutes, followed by approximately 200 ml of 4% paraformaldehyde in PBS over ~15-20 minutes The brain was extracted and incubated in 4% formaldehyde solution for 48 hr. After post-fixation, the brain was stored at 4°C in 1% PBS with 0.01% sodium azide. On the day of MR scanning, the brain was brought to room temperature, blotted dry, submerged in Fluorinert (3M, FC-3283), and embedded in a 15 mL tube, secured with gauze. The brain was imaged at room temperature in an 11.7T Bruker BioSpec Avance III preclinical MR system (Bruker BioSpin, Ettlingen, Germany) using a birdcage coil (T11232V3, transmitter, Bruker Biospin) combined with a mouse brain surface coil (T11657v3, receiver, Bruker Biospin). T1, T2, and Diffusion Weighted Images (DWI) were acquired (see Supp Table 4 for details). The temperature of the brain was monitored with a temperature probe (Small Animal Instruments Inc, NY, USA). The Bruker MR data were converted with Brkraw. The DTI data were corrected for distortions with FSL’s (FMRIB Software Library v6.0) eddy and the diffusion parameters were modelled with DTIFIT.

After MR scanning, the brain was washed in PBS and cryoprotected by immersing it in 30% sucrose in PBS at 4 ◦C until the brain sunk. The brain was fast-frozen on dry ice and sliced on a freezing microtome (Microm HM 440E Microtome) with a thickness of 30 µm. The brain slices were mounted using the mounting medium polyvinylpyrrolidone. A Zeiss Axio Imager A2 microscope upgraded with a stationary polarizer, quarter-wave plate, and rotating polarizer was used to acquire PLI images by alternating the angle of the birefringence of the tissue at nine systematic discrete equidistant angles from 0° to 160°. Together with a 2.5× magnifying objective, this yielded a spatial resolution of 1.76 μm/pixel. Background images were taken and used to correct for inconsistencies in illumination. Dispersion, retardance, and fiber orientation maps were created as described elsewhere. Per tissue section, a grid of high magnification images was taken, which were stitched together to create images of full brain slices using MATLAB (The MathWorks Inc., 1994–2017).

**Tracing**

Three adult females and one adult male *P. discolor* were used for tracing experiments. The surgical procedures are described in detail in previous publications^4,5^. Details of the stereotaxic device and the procedure used to reconstruct the recording sites are described elsewhere (Schuller1986). Briefly, the alignment of the animal’s skull and the underlying brain within the stereotaxic coordinate system was measured by scanning the characteristic profile lines of the skull in the parasagittal and frontal planes. These profiles were then digitally fitted to a standardized skull profile in a standardized coordinate system. The anaesthesia was antagonized with a mixture of atipamezole (Alzane®, Novartis), flumazenil (Flumazenil, Hexal) and naloxone (Naloxon-ratiopharm®, Ratiopharm), which was injected subcutaneously (2.5, 0.5 and 1.2 µg/g body weight, respectively). The bats were treated with antibiotics (0.5 µg/g body weight; enrofloxacin, Baytril®, Bayer AG) and an analgesic (0.2 µg/g body weight; Meloxicam, Metacam, Boehringer-Ingelheim) to alleviate postoperative pain for four postoperative days.

To determine the neural circuitry innervating the frontal auditory field (FAF), a neuronal tracer (18.2 nl of 10% Dextran Alexa Fluor 488, 10.000 MW, Thermo Fisher Scientific, Waltham, MA, USA) was pressure-injected (Nanoliter 2010 injector, World Precision Instruments, Sarasota, FL, USA) into the FAF. The injection site was electrophysiologically validated. After a survival time of 5 days, the animals were sedated with MMF (medetomidine (Dorbene®, Zoetis), midazolam (Dormicum®, Hoffmann-La Roche) and fentanyl (Fentadon®, Albrecht) at a dosage of 0.4, 4.0 and 0.04 µg/g body weight, respectively), euthanised with an intraperitoneal injection of pentobarbital (Narcore®, Boehringer Ingelheim Vetmedica GmbH, 0.16 mg/g bodyweight) and perfused transcardially by injecting approximately 200 ml of phosphate-buffered saline (PBS; pH 7.4) with 0.9% NaCl and Liquemin (with pH of 7.4) for 10 minutes, followed by approximately 200 ml of 4% paraformaldehyde in PBS over ~15-20 minutes. The brains were extracted and cryoprotected in 30% sucrose solution. They were then embedded (Schuller1986) and sliced to 40 µm thickness using a sliding microtome (Microm HM440E, Thermo Fisher Scientific). The slices were mounted (VECTASHIELD®HardSet™ Antifade Mounting Medium H-1400) and digitised (10 times magnification, 20 layer Z-stack, Axio Scan.Z1, Carl Zeiss Microscopy GmbH, Jena, Germany). Successfully labelled fibres and neurons were identified manually by observation of the images and their position was determined by comparing the results to a histological atlas of *P. discolor* brain (Radtke-Schuller et al., 2020.).

**Histological gene expression mapping**

One adult male bat was euthanised and the brain extracted as per the previous section. The brain was submerged in 4% paraformaldehyde for 48 hours and in PBS for 21 hours. Afterwards, the brain was washed with 1x PBS and cryoprotected via incubation in 30% sucrose (in PBS) at 4⁰C in a tube rotator until it sank in the solution (~overnight). The brain was dissected at the midline and each hemisphere was embedded in paraffin and sliced into sections of 4 μm thick. The section slices were placed onto Superfrost Plus glass microscope slides (*Thermo Scientific*) and baked in an oven at 57°C for one hour. The slides were stored in a dry box at room temperature until used.

For Nissl staining, the slides went through a series of washes with xylene, decreasing concentrations of ethanol (100%, 95%, 70 % ethanol), water and 0.1% cresyl violet. Slides were mounted with Dibutylphthalate Polystyrene Xylene mounting medium (DPX; Sigma). For immunohistochemistry, slides were deparaffinized and rehydrated with a series of washes of xylene, decreasing concentrations of ethanol (95, 70 and 50 % ethanol), and water. An antigen retrieval step was performed with homemade Tris-EDTA buffer (pH 9) in a microwave for five minutes at 840W and ten minutes at 210W. The slides were cooled with running water and submerged in 0.3% peroxidase solution for 30 minutes. Slides were washed with demiwater and the tissue on the slide was encircled with a PAP pen and blocked for one hour at room temperature with Normal Horse serum diluted to 10% in 1x PBS. The blocking solution was then replaced with a mix of blocking solution and primary antibody (see Supp Table 5) and incubated at 4°C overnight. Slides were then washed with 1x PBS and incubated with the secondary antibody for one hour at room temperature (see Supp Table 5). Next, slides went through an Avidin/Biotin (VECTASTAIN Elite ABC-HRP Kit PK-6100) step for amplifying the targeted antigen for 45 minutes. Slides were washed with 1x PBS and stained with 400 μl per slide of 3,3′-Diaminobenzidine solution (DAB VWRKBS04-500 from Immunologic). Slides were then dehydrated with washes of water and ethanol of increasing concentrations. Finally slides went through 2 xylene washes of 5 minutes each and mounted on cover slips with DPX mounting medium.

The identification of brain regions was done by comparing slides stained with Nissl, the *P. discolor* histology atlas^6^ and a mouse atlas^7^ for reference. Details of the antibodies are given in Supp Table 5. We performed control experiments without primary and secondary antibodies to test for nonspecific staining (data not shown). The FoxP1 and FoxP2 antibodies used in this study have been previously employed in studies on bats and their specificity was confirmed using other antibodies targeting different epitopes of the same gene (see Rodenas-Cuadrado et al.^8^ for specificity testing). We have not used the PV and GRIA1 antibodies in bats before, but the expression pattern is similar to what is expected based on observations in mice, supporting their specificity.

The stained sections were imaged using the Zeiss AxioScan.Z1 microscope in the Zeiss software ZEN using an in-house protocol for brightfield imaging at 20x magnification and a resolution of 0.22 μm per pixel, saved as .czi files, and exported as .tif files for analyses. The figure was constructed using Photoshop CC 2015 (Adobe). The schematic in panel A was drawn using the Nissl and protein expression images as guidelines. The area with no signal surrounding the stained slices was removed. To make the staining signal more visible on screen, the brightness of the full tissue images were adjusted using the 'levels’ parameter : specifically the midtones parameter of the image with the Nissl stain (panel B) was changed from 1.00 to 0.5 and the midtones parameter of the images with protein staining (panels C to F) was changed to 0.3. Original images can be shared upon request.

**PART III**

**Transgenic vector design and viral particle generation**

We designed a cassette to overexpress *P. discolor* FoxP2 with a fluorescent marker (GFP) that could be packaged into AAV5 viral particles to allow targeted transgene delivery in the bat brain. We designed the construct to expresses GFP and FoxP2 from the same promoter with a T2A element to allow expression of separate proteins whilst minimising vector size, given the packaging constraints of AAVs. The human Ubiquitin promoter (hUBC) was chosen as promoter in this cassette given the small size and previous pilot studies that showed robust expression from this promoter in the *P. discolor* brain (unpublished data). To facilitate detection, we added a series of small peptidic fusion tags (HIS-tag, FLAG-tag, T7, Xpress®) to FoxP2. To stabilize the transcript and enhance expression we used the human growth hormone (hGH) termination and polyadenylation signal. Production of the construct and viral particles were performed by Virovek using proprietary technology and the complete sequence is available upon request.

**Western blotting**

5.0 × 10^5 HEK293T/17 cells (ATCC, CRL-11268) were seeded in each well of a 6-well plate (70-80% confluence) 18-24 h prior to transfection of the plasmid used for viral packaging containing the full AAV5-GFP-FoxP2 cassette. 1 ug of DNA were used in the transfection alongside 3 ul of GeneJuice (Novagen, C134443) transfection reagent following the manufacturer protocol. Forty-eight hours post-transfection, cells were lysed in lysis buffer (0.1 M Tris, 150 mM NaCl, 10 mM EDTA, 0.2% Triton X-100, 1% PMSF, protease inhibitor cocktail) while rotating for 30mins at 4 °C and centrifuged at 10 000 g for 30 min at 4 °C, allowing cell debris to be pelleted and discarded. Western blotting was performed as described previously^9^. Proteins were detected using primary antibodies for 60 min at room temperature or at 4 °C overnight. Secondary antibodies were applied for 60 min at room temperature. Antibodies were used as follows: FoxP2 (Santacruz, sc-21069) at 1/500 concentration, β -actin (Sigma, A5441) at 1/1000 concentration, secondary goat-anti-mouse HRP-conjugated antibody (Bio-rad, 170-6516) or rabbit-anti-goat HRP-conjugated (DAKO, P0449) at 1/2000 concentration. The signal was developed using the Pierce ECL kit and detection was performed using the Imagelab 5.2.1 (Bio-Rad) on the Chemidoc system (Bio-Rad).

**Immunofluorescence in cells**

1.0 × 10^5 HEK293T/17 cells (ATCC, CRL-11268) were seeded on poly-L lysine coated glass coverslips in each well of a 24-well plate (70-80% confluence) 18-24 h prior to transfection of the plasmid used for viral packaging containing the full AAV5-GFP-FoxP2 cassette. 250ng of DNA were used in the transfection alongside 0.75ul of GeneJuice (Novagen, C134443) transfection reagent following the manufacturer protocol. 48hrs post-transfection the cells washed 2 times with 1x PBS (Lonza, BE17-516F) and fixed with 4% PFA for 6 minutes. After 2 rinses with 1x PBS to remove any traces of PFA, the cells were washed 3 times with IF wash buffer (0.1% Triton X-100 in TBS). Primary antibodies against FoxP2 (N-16 Santacruz, sc21069) and GFP (B2, Santacruz, sc9996), both at 1:200 dilution, were mixed in IF-block solution (1% Fish Gelatine, 0.1% Triton X-100, 5% BSA in PBS) and applied on the coverslip for an overnight incubation at 4 °C with gentle agitation. Following 4 washes with the IF-wash solution, the secondary fluorescence conjugated antibodies were mixed and applied to the coverslip for a 1hr incubation at room temperature with gentle agitation. The following secondary antibodies were used: donkey anti-goat IgG (H+L) Cross-Adsorbed Secondary Antibody Alexa Fluor 594 (A11058, Life Technologies) for FoxP2 and rabbit anti-mouse IgG (H+L) Cross-Adsorbed Secondary Antibody Alexa Fluor 488 (A11059, Life Technologies) for GFP, both at 1:500 dilution. The coverslips were washed 3 times with the IF-wash solution and one more time with the IF-wash solution to which Hoechst 33342 (Invitrogen, H3570) at a dilution of 1:10,000 was added. The stained coverslips were dipped in water to wash of the IF-wash solution and mounted on glass slides using fluorescent mounting medium (Dako, S3023) and then digitized (20 times magnification, Axio Scan.Z1, Carl Zeiss Microscopy GmbH, Jena, Germany). Antibody details are summarised in Supp Table 5.

**In vivo manipulation of FoxP2 expression**

To upregulate FoxP2 expression in the bat striatum *in vivo*, the viral construct (AAV5-hUBC_GFP_T2A_TAGS_FOXP2_hGH_PA_term, 2.0E13 vg/ml, 600 nl per injection site) was pressure-injected (Nanoliter 2010 injector, World Precision Instruments, Sarasota, FL, USA) into the striatum of two adult males, following the surgical and stereotaxic protocol from the tracing injections (above). A total of 4 injections were administered in a rostro-caudal line, starting at 4000 µm from the onset of the neocortex and spaced by 500 µm. The line of injections was located 1500 µm laterally from the midline and the injection depth was ~3500 µm, respectively. To facilitate within-animal comparisons of the efficacy of the transgene, a control virus expressing only the GFP protein (AAV5-CMV-GFP, 2.0E13 vg/ml, 600 nl per injection site) was injected into the opposing hemisphere in an identical pattern. After an incubation time of 10 days, the animals were sedated, euthanised and the brains were extracted following transcardial perfusion (as per Part I).

**Immunofluorescence of transgenic bat brains**

Brains dissected from animals injected (as per methods from Part I) were cryoprotected in 30% sucrose solution for 48 hours until they started sinking in the solution. The brains were then embedded in OCT Compound (Sakura) and sliced into 8µm thick sections and transferred to Superfrost plus gold glass slides (Menzel Gläser). The slides were stored at -80 degrees. The staining to reveal the FoxP2 expression was performed as follows; the slides were fixated in 4% formaldehyde solution and washed in PBS three times for 5 minutes. Antigen retrieval was performed in pH 9 Tris‐EDTA buffer (homemade) in a waterbath for 1 hour minimium at 65 degrees. The tissue was then encircled with a PAP pen and blocked with 10% normal serum (Vector labs) for 1 hr at room temperature. After blocking, the slides were incubated overnight at 4°C with primary antibodies diluted in appropriate blocking solution (see Supp Table 5 for an overview); the antibody against FoxP2 (Merck, MABE415) was used at a dilution of 1:500 and the antibody against GFP (GFP I-16, Santa Cruz, sc-5385) was used at a dilution of 1:250. The following day, sections were washed three times with PBS and incubated with the corresponding secondary antibody for 1 hr at room temperature. Slides stained for FoxP2 were first incubated in the appropriate secondary conjugated with biotin, washed three times in PBS and afterwards incubated for one hour in streptavidin conjugated to Alexafluor A647 (Invitrogen) and washed three times in PBS. To detect GFP, the secondary antibody donkey anti goat A488 (Invitrogen, A-11055) at 1:200 was used. Slides were then treated with a Hoechst (DAPI) stain diluted 1:10,000 in PBS for 10 minutes, washed in PBS and mounted using DAKO fluorescent mounting media (Dako, S3023). The slides were sealed using clear nail polish and imaged using a Zeiss Axio Scan.Z1 (Carl Zeiss Microscopy GmbH, Jena, German). Antibodies details are summarized in Supp Table 5.

**Image Analysis and Quantification**

Images were exported to TIFF format using the Zen software (Carl Zeiss Microscopy GmbH, Jena, Germany). In order to quantify the signal intensity, regions of interest within the infected area of the same size (3000 pixels x 3000 pixels) were extracted from each hemisphere in the striatum of *P. discolor* from two different slides derived from separate experiments. The regions of interest were exported in TIFF format, with one file for each channel individually (DAPI – for the nuclei count, GFP to delimit the infected area, FoxP2 to count positive cells and quantify expression). These files were then analysed using MetaMorph (Molecular Devices, LLC, San Jose, CA 95134 USA) image analyses tools. The DAPI and FoxP2 channels were analysed with the count nuclei function using the following parameters: Minimum width: 5, Maximum width: 15, Gray level above background: 20. The data were exported into excel files for control and experimental hemisphere respectively and saved as CSVs. CSVs were then imported in R-Studio and the integrated intensity measures and the cell count data were used for plotting and statistical analyses (T-test).

**SUPPLEMENTARY REFERENCES**

1 Zawada, A. M. *et al.* Massive analysis of cDNA Ends (MACE) and miRNA expression profiling identifies proatherogenic pathways in chronic kidney disease. *Epigenetics* **9**, 161-172, doi:10.4161/epi.26931 (2014).

2 Jebb, D. *et al.* Six reference-quality genomes reveal evolution of bat adaptations. *Nature* **583**, 578-584, doi:10.1038/s41586-020-2486-3 (2020).

3 Hackenberg, M., Rodriguez-Ezpeleta, N. & Aransay, A. M. miRanalyzer: an update on the detection and analysis of microRNAs in high-throughput sequencing experiments. *Nucleic Acids Res* **39**, W132-138, doi:10.1093/nar/gkr247 (2011).

4 Hoffmann, S. *et al.* Psychophysical and neurophysiological hearing thresholds in the bat Phyllostomus discolor. *J Comp Physiol A Neuroethol Sens Neural Behav Physiol* **194**, 39-47, doi:10.1007/s00359-007-0288-9 (2008).

5 Horpel, S. G. & Firzlaff, U. Processing of fast amplitude modulations in bat auditory cortex matches communication call-specific sound features. *J Neurophysiol* **121**, 1501-1512, doi:10.1152/jn.00748.2018 (2019).

6 Radtke-Schuller, S., Fenzl, T., Peremans, H., Schuller, G. & Firzlaff, U. Cyto- and myeloarchitectural brain atlas of the pale spear-nosed bat (Phyllostomus discolor) in CT Aided Stereotaxic Coordinates. *Brain Struct Funct* **225**, 2509-2520, doi:10.1007/s00429-020-02138-y (2020).

7 Lein, E. S. *et al.* Genome-wide atlas of gene expression in the adult mouse brain. *Nature* **445**, 168-176, doi:10.1038/nature05453 (2007).

8 Rodenas-Cuadrado, P. M. *et al.* Mapping the distribution of language related genes FoxP1, FoxP2, and CntnaP2 in the brains of vocal learning bat species. *J Comp Neurol* **526**, 1235-1266, doi:10.1002/cne.24385 (2018).

9 Devanna, P. *et al.* Next-gen sequencing identifies non-coding variation disrupting miRNA-binding sites in neurological disorders. *Mol Psychiatry* **23**, 1375-1384, doi:10.1038/mp.2017.30 (2018).
